# CAULIFINDER: a pipeline for the automated detection and annotation of caulimovirid endogenous viral elements in plant genomes

**DOI:** 10.1186/s13100-022-00288-w

**Published:** 2022-12-03

**Authors:** Héléna Vassilieff, Sana Haddad, Véronique Jamilloux, Nathalie Choisne, Vikas Sharma, Delphine Giraud, Mariène Wan, Saad Serfraz, Andrew D. W. Geering, Pierre-Yves Teycheney, Florian Maumus

**Affiliations:** 1 Université Paris-Saclay, INRAE, URGI, 78026 Versailles, France; 2 Present Address: Service d’Etude des Prions et des Infections Atypiques (SEPIA), Institut François Jacob, Commissariat à l’Energie Atomique et aux Energies Alternatives (CEA), Université Paris Saclay, Fontenay-aux-Roses, France; 3 Present Address: Université Paris-Saclay, INRAE, PROSE, 92160 Antony, France; 4 Present Address: Forschungszentrum Jülich GmbH, Institute for Bio- and Geosciences 1, IBG1, 52425 Jülich, Germany; 5 UMR AGAP Institut, Univ. Montpellier, CIRAD, INRAE, Institut Agro, 20230 San Giuliano, France; 6 CABB, University of Agriculture Faisalabad, Faisalabad, 38000 Pakistan; 7 Queensland Alliance for Agriculture and Food Innovation, The University of Queensland, St Lucia, QLD 4072 Australia; 8 CIRAD, UMR PVBMT, F-97410 Saint Pierre, La Réunion France

**Keywords:** Endogenous viral elements, Plant genomes, Paleovirology, *Caulimoviridae*, Repetitive elements, Bioinformatics, Genome annotation

## Abstract

Plant, animal and protist genomes often contain endogenous viral elements (EVEs), which correspond to partial and sometimes entire viral genomes that have been captured in the genome of their host organism through a variety of integration mechanisms. While the number of sequenced eukaryotic genomes is rapidly increasing, the annotation and characterization of EVEs remains largely overlooked. EVEs that derive from members of the family *Caulimoviridae* are widespread across tracheophyte plants, and sometimes they occur in very high copy numbers. However, existing programs for annotating repetitive DNA elements in plant genomes are poor at identifying and then classifying these EVEs. Other than accurately annotating plant genomes, there is intrinsic value in a tool that could identify caulimovirid EVEs as they testify to recent or ancient host-virus interactions and provide valuable insights into virus evolution. In response to this research need, we have developed CAULIFINDER, an automated and sensitive annotation software package. CAULIFINDER consists of two complementary workflows, one to reconstruct, annotate and group caulimovirid EVEs in a given plant genome and the second to classify these genetic elements into officially recognized or tentative genera in the *Caulimoviridae*. We have benchmarked the CAULIFINDER package using the *Vitis vinifera* reference genome, which contains a rich assortment of caulimovirid EVEs that have previously been characterized using manual methods. The CAULIFINDER package is distributed in the form of a Docker image.

**Supplementary Information:**

The online version contains supplementary material available at 10.1186/s13100-022-00288-w.

## Introduction

The *Caulimoviridae* is the only family of plant viruses with a double-stranded DNA (ds) genome. Some of its members cause serious diseases, such as rice tungro and cacao swollen shoot [1, 13]. Eleven genera are currently recognized in the family *Caulimoviridae* by the International Committee on Taxonomy of Viruses (ICTV), based on differences in genome organization, virion morphology, replication strategy and mode of vector transmission [38]. Genome sizes range from 6.9 to 9.8 kbp and the number of open reading frames (ORFs) is very variable, from a single ORF encoding a large polyprotein in the case of petunia vein clearing virus (PVCV) in the genus *Petuvirus*, to nine ORFs for rose yellow vein virus in the genus *Rosadnavirus*. The dsDNA genomes of members of the *Caulimoviridae* are non-covalently closed, circular molecules but are typically represented in a linear form and the first nucleotide of the tRNA^Met^ motif designated the beginning of the genome sequence [38].

The *Caulimoviridae* is one of five families in the order *Ortervirales*, which also includes the *Retroviridae*, *Pseudoviridae*, *Metaviridae* and *Belpaoviridae* [21]. All families in this order share a core *Gag*-*Pol* gene cassette. The Gag (group antigen) proteins are involved in virion assembly, whereas the Pol (polymerase) polyprotein is processed into aspartyl protease (AP) and reverse transcriptase (RT) enzymes, the latter having a tethered ribonuclease H1 (RH1) domain. To this rudimentary replication unit are added different auxiliary genes that allow the viruses to occupy different ecological niches, such as movement protein (MP), virion-associated protein and aphid transmission factor genes in the case of the *Caulimoviridae*, which facilitate systemic infection and then vector transmission in plants [17, 27, 37]. Pol polyproteins are highly conserved at primary, secondary and tertiary structural levels and therefore utilized for classification from order to species levels. The *Caulimoviridae* is a sister taxon to the *Metaviridae*, which includes the so-called *Gypsy*-like long terminal repeat (LTR) retrotransposons [21, 24, 41]. The other ubiquitous group of *Ortervirales* in plants is the *Copia*-like LTR retrotransposons and they are classified in the *Pseudoviridae* [25].

Members of the *Caulimoviridae* do not actively integrate in their host genome as part of their replication cycle and are therefore often referred to as plant-infecting pararetroviruses. Nevertheless, caulimovirid endogenous viral elements (EVEs), sometimes referred to as endogenous pararetroviruses (EPRVs), are widespread across the plant kingdom [7, 12, 19, 20]. It is thought that this DNA has been captured in the plant genome either by non-homologous or microhomology-mediated end-joining during double-stranded DNA repair or by using hybrid integration mechanisms involving transposable elements (TEs) [12, 22]. Many caulimovirid EVEs exist at very high copy numbers and this may result from repetitive integration of the same virus during one or more bouts of infection and/or amplification of this sequence by cycles of activation and reintegration [31].

Using RT gene sequences of extant *Caulimoviridae* to scan plant genome and transcriptome databases for the presence of EVEs, a dozen operational taxonomic units (OTUs) have been identified that appear to exist only in the form of EVEs. These include the tentative genus Florendovirus, which is widespread in the genomes of flowering plants, as well as four different tentative Gymnendovirus and two Fernendovirus genera that are found in gymnosperm and fern genomes, respectively [7, 12]. Although caulimovirid EVEs have been identified in a wide range of tracheophyte plants [7], their potential impact on plant genome evolution remains largely unknown. However, integration and/or amplification and deletion of caulimovirid EVEs has the potential to impact both the structure and function of host genes through insertional mutagenesis or by altering the level or tissue specificity of transcription of nearby host genes through the capture of novel viral promoter regulatory elements [33]. Furthermore, the presence of an EVE likely affects the epigenetic landscape around that EVE.

Most integration events are followed by sequence decay and result in replication-defective EVEs. However, as the mutations tend to be random in distribution, it is possible to assemble complete or near complete consensus sequences in silico that reflect the ancestral viral genomes. This approach has provided insights into the long-term evolution of the *Caulimoviridae*, including evidence for the development of bipartite genomes [7, 12].

The discovery and then characterization of caulimovirid EVEs currently relies upon manual search and assembly methods, which are time-consuming, therefore limiting the amount of data that can be processed, and also highly skills-dependent. This approach cannot keep up with the exponential growth of plant genome sequence data. There is a need for a standardized and more efficient annotation procedure allowing plant genomes to be scanned rapidly and accurately to detect these genetic elements. Importantly, there are often large and diverse sequence populations of caulimovirid EVEs in plant genomes and using non-purpose-built methods of identification, these appear as dispersed repetitive elements. Repeat annotation programs like RepeatModeler [34] or the TEdenovo pipeline from the REPET package [11] can produce consensus sequences that represent caulimovirid EVEs but these programs inherently search for all interspersed repeats. The available tools to classify these consensus sequences, for example PASTEC [16], often misclassify caulimovirid EVEs as LTR retrotransposons. Lastly, low copy caulimovirid EVEs are not normally detected by repeat detection programs and this error can be consequential, as some EVEs are present at only single genome loci but are still capable of being reactivated to cause new infections [6].

In this paper, we describe a new software pipeline called CAULIFINDER, which has been designed to annotate and classify caulimovirid EVEs in plant genomes using homology-driven approaches. The use of CAULIFINDER is demonstrated using the *Vitis vinifera* reference genome, which contains a diversity of well-characterized caulimovirid EVEs, allowing the benefits and limitations of this new genome annotation tool to be assessed.

## Implementation

CAULIFINDER consists of two complementary pipelines: Branch A reconstructs consensus sequences representative of repetitive caulimovirid EVEs and Branch B collects representative sequences and classifies them into officially recognized or tentative genera of the *Caulimoviridae*. Branch B is also capable of detecting low copy number caulimovirid EVEs.

### Description of Branch A – sequence retriever

**Fig. 1:**
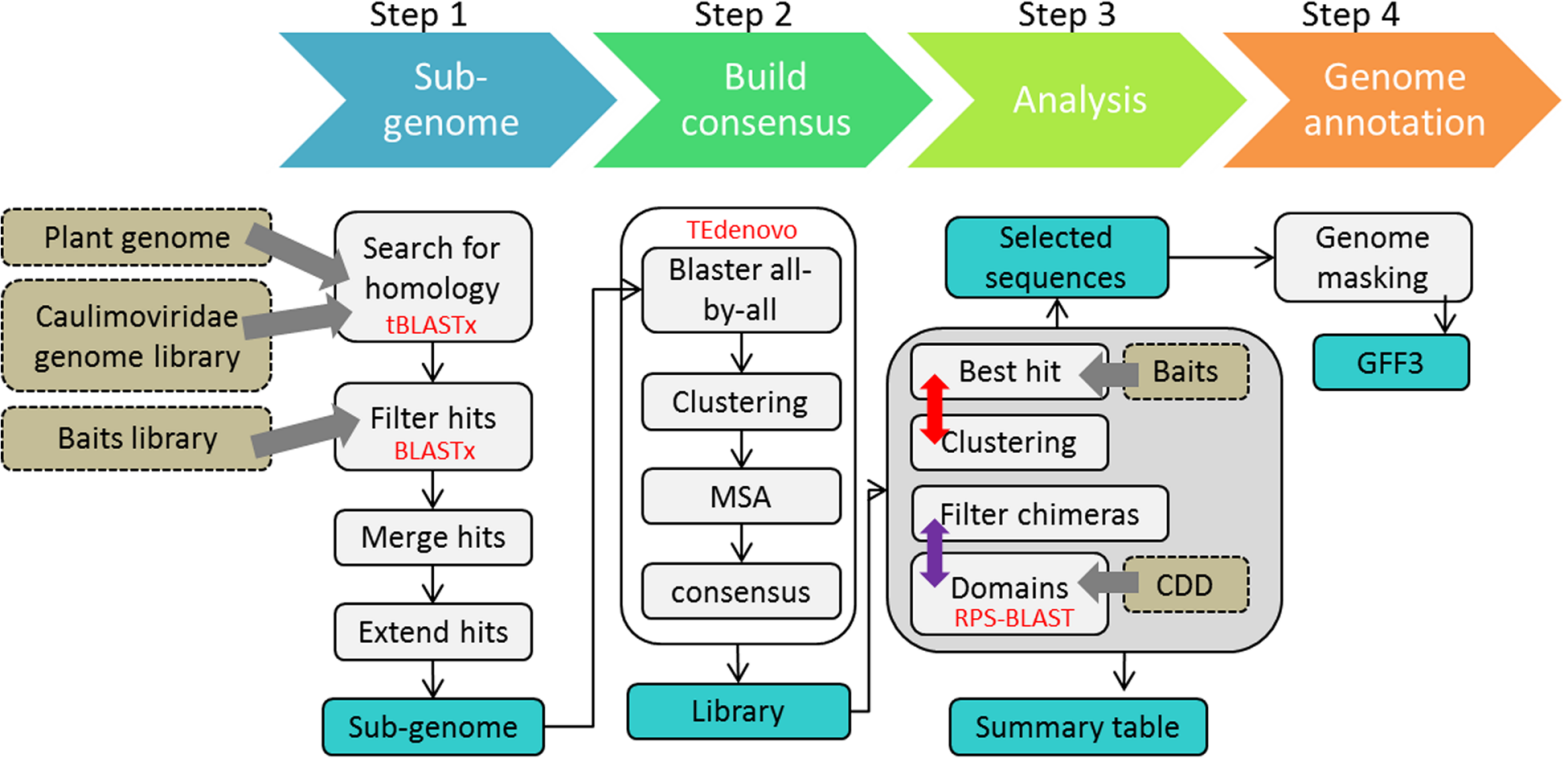
Overview of CAULIFINDER Branch A workflow. The line of arrows at the top represents the four main steps of the workflow. Grey boxes indicate successive sub-steps with the main tools highlighted in red font. The dark grey box comprises several analyses that are run on the consensus library. The main output files are shown in blue boxes. The input datasets are shown in khaki boxes with arrows indicating in which sub-step they are used. The red two-headed arrow represents the “blastclust_supplementation” option (default = FALSE). The purple two-headed arrow represents the “filter_chimeras” option (default = TRUE)

CAULIFINDER Branch A aims to construct a library of consensus sequences that is representative of all repetitive caulimovirid EVEs present in a plant genome and which is devoid of contaminating LTR-retrotransposon sequences. This library typically contains complete and near complete virus genomes, as well as fragments and concatemers of these genomes (see below). Branch A creates clusters of consensus sequences sharing high nucleotide sequence similarity in order to establish relationships between the consensus sequences. This function also provides the capacity to group different components of a putative multipartite genome and connect fragments of sequence with longer, more complete sequences. The four steps of the workflow are described in detail below and summarized in Fig. 1.

#### Step 1- construction of a genomic subset

This first step of Branch A aims to identify putative endogenous caulimovirid EVE loci by doing a tBLASTX [3] search of a plant genome database using a reference library of caulimovirid genomes as the query sequences. This library comprises 11 genome sequences representing each exemplar virus isolate of the officially recognized genera in the *Caulimoviridae*, as well as reconstructed genome sequences of each of the 12 tentative genera of caulimovirid EVEs proposed by [7]. The current library is described in Supplementary Table 1 and the latest version will be available in a dedicated dataverse (10.57745/ADFNMB). After tBLASTx against the plant genome, overlapping hits are merged and then extracted from the genome database as FASTA-formatted sequences. Due to the conserved nature of the RT domain in particular, many hits are distantly related *Copia* and *Gypsy*-like LTR retrotransposons (*Metaviridae* and *Pseudoviridae*), which are abundant in plant genomes. To filter out these sequences, extracted sequences are compared by BLASTX alignment (BLAST+ package [3] to a library called “baits.fa”, which contains a comprehensive set of caulimovirid protein sequences as well as the collection of RT and RNaseH protein sequences from *Copia*- and *Gypsy*-like LTR retrotransposons from the Gypsy database (https://gydb.org/) [23]. Those sequences with best hits to the retrotransposon proteins are discarded. The remaining sequences that map to approximately the same location in the plant genome (separated by 5 kbp or less) are then merged, since these loci probably derive from single integration events. This merging process consolidates different hits obtained for the same locus. The loci are then extended in both directions (option -X: “extension value”, 2 kbp by default) to capture any remaining ORFs and non-coding sequences and identify the junctions of the EVE with the plant DNA. Finally, the extended loci are extracted from the plant genome database as FASTA-formatted sequences and combined into a single file to build a genomic subset enriched for caulimovirid EVE loci (hereafter referred to as “sub-genome”). This enrichment process allows more sensitive search parameters to be applied in the next step without a cost in computational time compared to whole genome repeat identification.

#### Step 2- building a consensus library

In the second step of Branch A, the sub-genome is used as input for the TEdenovo pipeline from the REPET package [11]. TEdenovo identifies high-scoring pairs (HSPs) using Blaster and these are used by three sequence clustering algorithms, Grouper, Piler and Recon [10, 29, 30], to identify groups of HSPs sharing high sequence similarity. By default, CAULIFINDER Branch A only considers the groups containing at least five HSPs (option -hsp: “HSP number”), meaning that at least five caulimovirid EVE sequences must group together. A maximum of 20 sequences from each group are then aligned to produce respective consensus sequences. Apart from reducing computational time, use of an enriched sub-genome dataset allows TEdenovo sensitivity to be increased as an all-by-all BLAST identity threshold of 85% can be used, which is not recommended for use on a whole genome.

#### Step 3- characterization of consensus sequences

In the third step of Branch A, each consensus sequence is scanned for conserved protein domains using RPS-BLAST against the CDD database [26] and those containing a TE-specific protein domain, for example, an integrase domain, are discarded (option -ch: “filter_chimeras” set to TRUE by default). Consensus sequences are also compared using BLASTx to the library “baits.fa” in order to discard potential false positives and to provide a preliminary classification to genus rank in the *Caulimoviridae*. In addition, consensus sequences are clustered on the basis of pairwise similarity and coverage using BLASTCLUST (ftp://ftp.ncbi.nih.gov/blast/documents/blastclust.html) to establish groups that share high sequence homology (options -S: “identity” and -L: “coverage”, set to 90% and 0.9 by default, respectively). Besides grouping redundant sequences, this last sequence sorting procedure is intended to identify components of a divided virus genome. By default, only the consensus sequences with best hits to caulimovirid protein sequences in the “baits” library are selected. However, to allow additional interrogation of the content of each cluster, an option allows selecting all the sequences from the clusters containing at least one consensus sequence with best hits to caulimovirid protein sequences (option -bl: “blastclust_supplementation” set to FALSE by default) (Fig. 1). It should be noted that the CAULIFINDER output library keeps all selected sequences without removing redundancy. This choice is intentional in order to retain all possible structural variants and all components of bipartite genomes.

#### Step 4- genome annotation

In the last step, the selected consensus sequences are used as a library to run the RepeatMasker program [35] against the whole plant genome of interest. This enables a genome-wide detection of the positions of the caulimovirid EVEs and provides a genome coverage estimate.

### Description of Branch B – marker miner

**Fig. 2:**
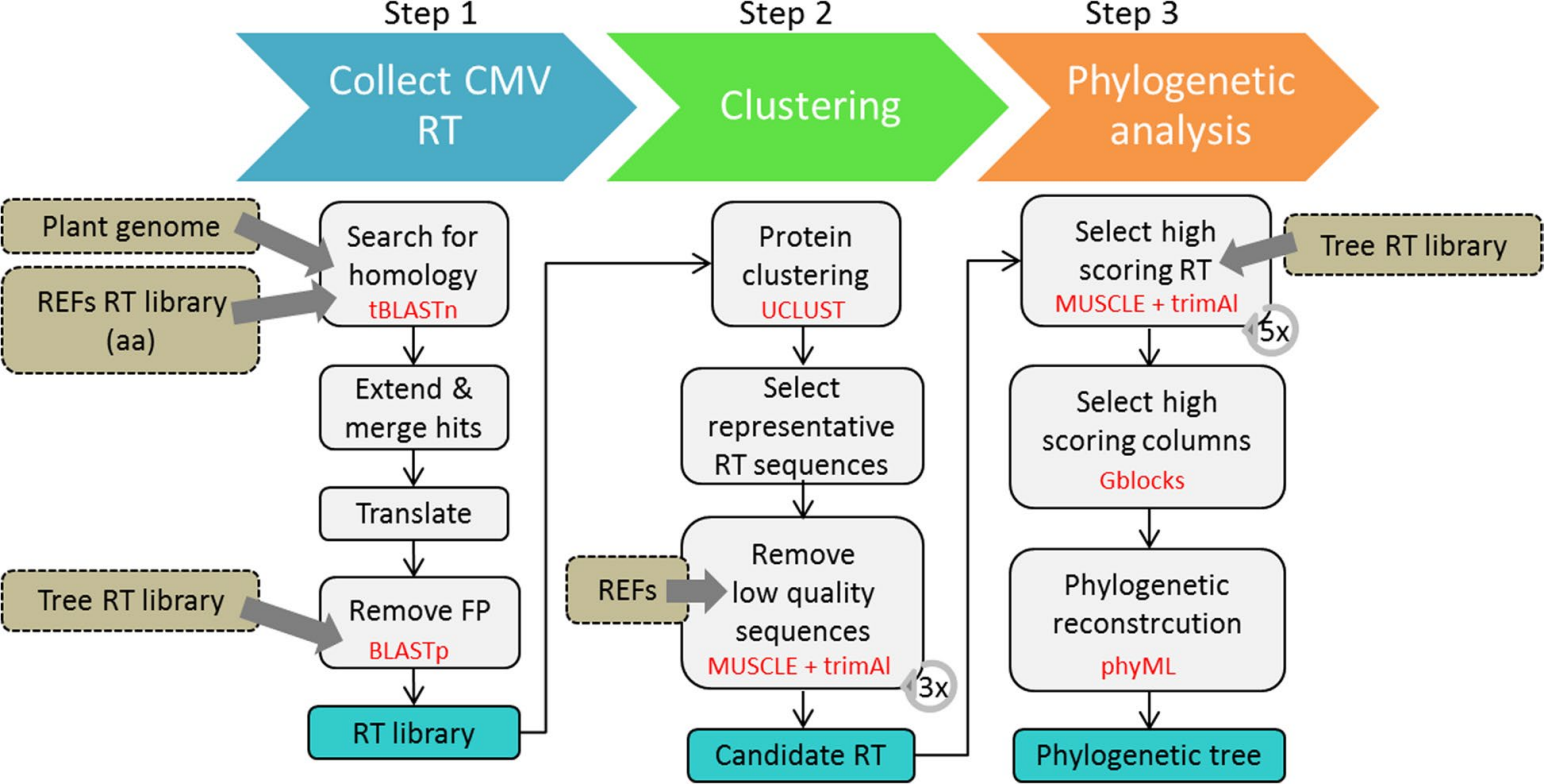
Overview of CAULIFINDER Branch B workflow. The line of three arrows on the top represents the three main steps of the workflow. Grey boxes indicate successive sub-steps with the main tools highlighted with red font. The main output files are shown in blue boxes. The input datasets are shown in khaki boxes with arrows indicating in which sub-step they are used. The grey looping arrows in steps 2 and 3 indicate the number of iterations of sequence selection using protein alignment with MUSCLE, followed by trimAl with empirical parameters

CAULIFINDER Branch B aims to extract the RT protein sequences from caulimovirid EVEs present in a plant genome and to use them in a phylogenetic analysis for the purpose of classification. The results using this approach are more accurate than those obtained by best hit from a BLAST search in Branch A, especially for EVEs representing novel genera. Branch B also detects low copy number and single-copy RT loci, whereas Branch A only recognizes repetitive elements. The three steps of the workflow are described in detail below and summarized in Fig. 2.

#### Step 1- collect caulimovirid EVE RT sequences

The workflow begins with a tBLASTn search of a plant genome for matches to a library of reference caulimovirid RT sequences. Hits to the target genome are extended 120 bp upstream and downstream to account for breaks in the BLAST alignment and overlapping regions are merged when found. The corresponding DNA sequences are then conceptually translated into protein sequences with a minimum length of 200 amino acids (aa) and compared to a library of RT protein sequences covering the *Caulimoviridae* and *Copia*- and *Gypsy*-like LTR retrotransposons using BLASTp. The plant genome RT protein sequences with the highest scoring hits to members of the *Caulimoviridae* are then selected.

#### Step 2- select workable RT protein sequences

In the second step of the workflow, selected protein sequences are sorted into groups using similarity-based clustering with UCLUST set at an 80% aa identity threshold [9]. One representative sequence per group is then selected and these are combined into the initial library of diverse caulimovirid RT sequences. This collection of sequences is aligned using MUSCLE [8] and sequences with quality scores below cutoff in the multiple sequence alignment are removed using trimAl [4]. Two additional rounds of alignment with MUSCLE followed by filtering using trimAl are run using empirical parameters to further remove poorly aligned RT sequences (Fig. 2). At the end of this step, the output file is generated, corresponding to representative caulimovirid EVE RT sequences found in the input genome.

#### Step 3- phylogenetic analysis

In the last step, the representative caulimovirid EVE RT sequences are combined to form a library that contains reference RT sequences from all *Caulimoviridae* genera (established from the work described in [7]) and outgroup sequences from retroviruses and LTR retrotransposons. This library is used to produce a multiple sequence alignment (MSA) using MUSCLE and sequences having high alignment quality are selected using trimAl. Four additional rounds of alignment with MUSCLE followed by filtering using trimAl are carried out (Fig. 2). This iterative process is meant to ensure high quality protein datasets necessary for automated phylogenetic reconstruction. The last MSA is curated using GBLOCKS [5] to remove poorly aligned sequence columns and then deliver this new file to PhyML [15] for phylogenetic reconstruction. The output tree is provided in Newick format and results can be visualized using a tree-visualization application such as iTOL (https://itol.embl.de/upload.cgi). With this automated phylogenetic analysis, Branch B allows a quick and straightforward assessment of the diversity of caulimovirid EVEs by providing tentative classifications to genus rank in the *Caulimoviridae*.

### Development and distribution

CAULIFINDER pipelines are coded in bash and python scripts (2.7) that are versioned as gitlab project saved in forgemia (https://forgemia.inra.fr/urgi-anagen/event_caulifinder), one of the forge provided by INRAE. The fasta libraries and the documentation to launch Branch A and Branch B are also available in this gitlab project. The fasta libraries are also published in a dataverse (10.57745/ADFNMB) where they will be updated and versioned.

As FAIR principles (Findable, Accessible, Interoperable and Reusable) [40] apply to research pipelines, we have decided to deploy the CAULIFINDER pipelines in the Docker open access container image delivery system [28] to meet the Open Science goals. Hence, CAULIFINDER can be used in various environments (cloud, serverless and operating systems like linux) and always under the same conditions (all dependencies in the same soft version) to ensure repeatability. Furthermore, this deployment strategy circumvents problems associated with the installation of dependencies. Users only need to install the container engine and launch genome analysis in a project directory. The CAULIFINDER container is available on dockerhub (https://hub.docker.com/r/urgi/docker_caulifinder) and its code is deposited in the public gitlab repository “Caulifinder_docker” (https://forgemia.inra.fr/urgi-anagen/caulifinder_docker) together with a content description and usage instructions.

The image is designed with:

- the Centos7 operating system
- Slurm as jobs scheduler
- MariaDB as database manager through the free docker image docker-centos7-slurm (https://hub.docker.com/r/giovtorres/docker-centos7-slurm) from dockerhub
- REPET instance v2.5
- other free softwares listed in a README file (https://forgemia.inra.fr/urgi-anagen/event_caulifinder/-/blob/master/readme)
- Caulifinder pipelines

The tests at pipeline level were done on benchmark using the 12X version of *Vitis vinifera* PN40024 reference genome sequence (assembly GCA_000003745.2 (https://www.ncbi.nlm.nih.gov/assembly/GCA_000003745.2) [18]). By default, Branch A and Branch B took 90 minutes and 7 minutes, respectively, to complete on a CentOS7 virtual machine with 32 vCPU and 64 Go of RAM. On another CentOS7 virtual machine (with 16 vCPU and 32 Go of RAM), Branch A took 99 minutes to complete.

## Results

We used the *Vitis vinifera* reference genome PN40024 [18] to benchmark CAULIFINDER because several caulimovirid EVE genomes have been manually reconstructed from this genome by an expert virus taxonomist, namely four tentative florendovirus species (VvinAV, VvinBV, VvinCV and VvinDV), of which two have putative bipartite genomes (components A and B of VvinBV and VvinDV). Using these consensus sequences as queries, endogenous florendoviruses were estimated to contribute 0.65% of the total *V. vinifera* genome content [12]. Additionally, Vitis endovirus, a representative of a novel genus, was identified by Diop et al. [7]. Complete or near complete viral genomes have been reconstructed for these caulimovirid EVEs, and each varies in copy number, sequence length and probably age of integration as evident by the number of mutations [12].

### Benchmark of Branch A

#### Run 1: default parameters and default libraries

To benchmark Branch A, we first assessed its capacity to build consensus sequences corresponding to the reference sequences of the different florendovirus species and Vitis endovirus. CAULIFINDER Branch A was launched with default parameters and default libraries on the *V. vinifera* PN40024 genome (486 Mb). At the end of step 1, a sub-genome of 19.7 Mb was obtained and at the end of step 2, 536 consensus sequences were assembled. At step 3, 144 consensus sequences were selected, representing 23 clusters. We noticed that all 144 selected sequences had best hits against either one of the florendovirus or Vitis endovirus sequences. Step 4 provided an estimate of 1.4% coverage of the *V. vinifera* genome by caulimovirid EVEs (Table 1). The main output files of this run are available in the supplementary dataset.

**Table 1:** Main metrics of Branch A runs performed on *V. vinifera*

	Run 1	Run 2	Run 3
Genome size (bp)	486,198,630	486,198,630	486,198,630
Sub-genome size (bp)	19,740,343	18,635,134	41,454,669
# of consensus (step 2)	536	519	1557
# of selected consensus (step3)	144	116	219
# of clusters	23	25	20
Genome coverage (%)	1.40	0.85	3.05
# of chimeras	0	0	0
Conserved domains in selected consensus (step 3)	MP protease RNaseH RT RT_RNaseH RT_RNaseH_2 RVP zf-CCHC ZnF_C2HC	MP protease RNaseH RT RT_RNaseH RT_RNaseH_2 RVP zf-CCHC ZnF_C2HC	MP protease RNaseH RNase_H_like RT RT_RNaseH RT_RNaseH_2 RVP zf-CCHC ZnF_C2HC

**Fig. 3:**
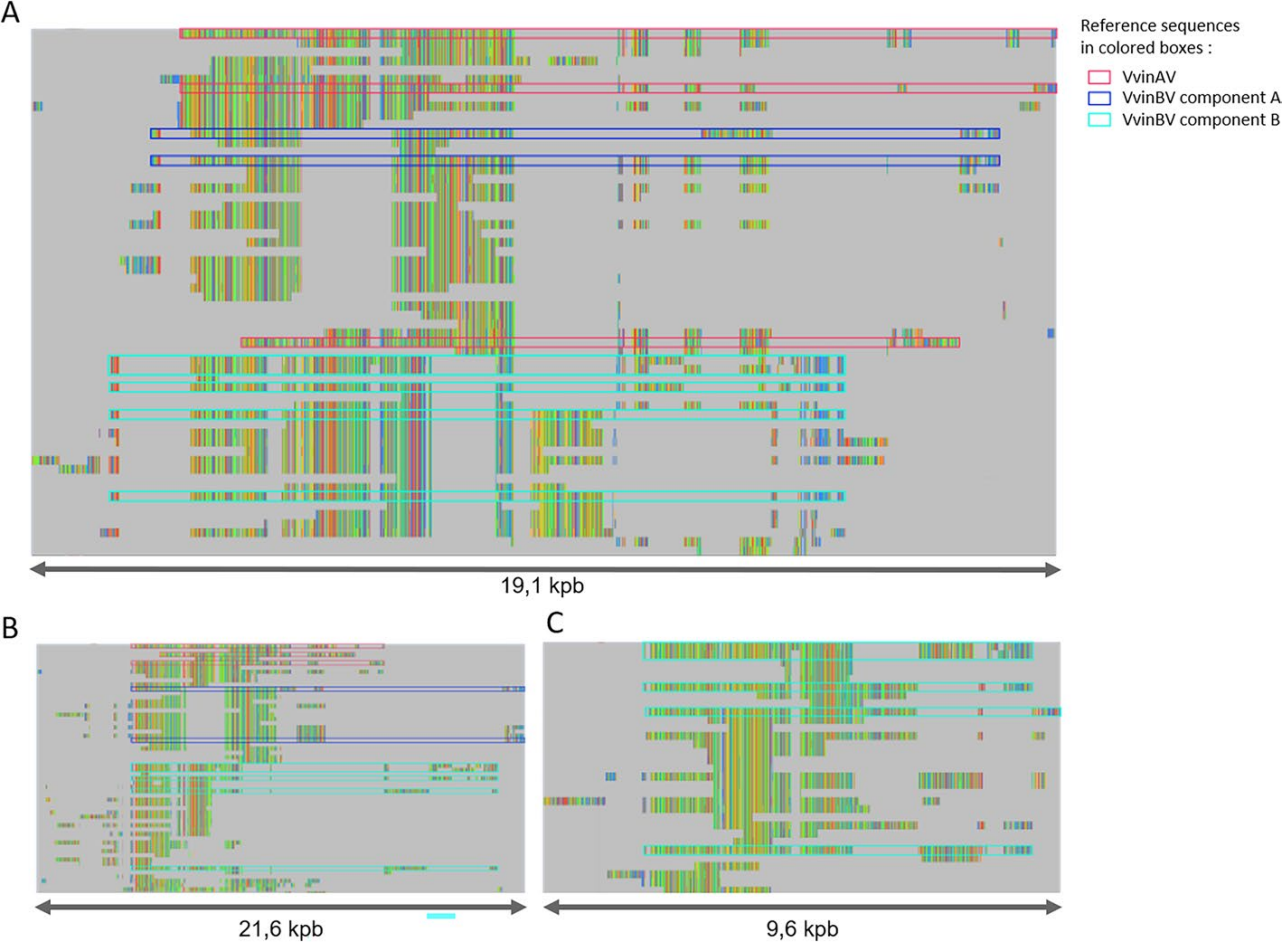
Overview of the multiple sequence alignments obtained for the VvinAV-VvinBV cluster using CAULIFINDER Branch A. The alignments were obtained using MAFFT with the ginsi and leave gappy regions 0.8 settings and visualized in the overview window of the Jalview program [39] with the following nucleotide colours: A (green), T (blue), G (red), C (orange). The colour densities are smoothed in the overview. The reference sequences are highlighted in red (VvinAV), blue (VvinBV_compA) or turquoise (VvinBV_compB). Branch A output sequences are not highlighted and concatemers have been removed for the ease of visualization. The raw alignments can be visualized in Supplementary Fig. 1. The alignments correspond to run 1 (**A**), run 2 (**B**) and run 3 (**C**). For the latter, only the VvinBV_compB sequences are shown

As a proxy for sensitivity, we next investigated the similarity of the CAULIFINDER output sequences with the 17 reference florendovirus and single Vitis endovirus sequences by combining all together in a single file and clustering alike sequences using 85% nt identity and 85% target sequence coverage parameters. All 17 reference sequences grouped in clusters containing at least one CAULIFINDER output sequence. In one sequence cluster, VvinAV and VvinBV were grouped together, a result that reflects their high sequence similarity [12], while VvinCV and VvinDV each formed separate sequence clusters. The two components of the bipartite genomes always grouped in the same sequence cluster. Additionally, reference sequences for each of the tentative virus species accurately sorted into the sequence cluster that contained its closest relatives. Fifteen CAULIFINDER output sequences grouped in clusters that did not contain reference virus sequences, of which 13 were singletons. All had best hits against one of the florendoviruses or Vitis endovirus, suggesting that the singletons represented poorly assembled or more divergent species among these genera. Cluster-wise sequence alignments revealed that the reference sequences from Vitis endovirus and all tentative florendovirus species were either completely or mostly embedded in at least one CAULIFINDER consensus sequence (Fig. 3A and Supplementary Fig. 1).

The lengths of three consensus sequences exceeded the length of the cognate reference virus sequences by over 10 kbp. These abnormally long sequences contained multiples of conserved protein domains (Table 1), suggesting that they represented concatemers of a virus genome, a fact that was confirmed by manual inspection (Supplementary Fig. 2). The construction of concatemers is not surprising since caulimovirid EVEs are often present as tandem repeats or cluster in genome hotspots [2, 12, 14, 32]. It is likely that the extension step performed on the merged hits favored sequences that are redundant to each merged hit. Suspected concatemeric sequences can be interrogated using dot plot sequence alignment tools, and duplicated sequences can then be manually removed to obtain a monomer. This manual curation is not necessary to run the RepeatMasker annotation (step 4) but should be done when reconstructing ancestral viral genomes.

Finally, we investigated whether CAULIFINDER consensus sequences might be contaminated by TE-derived sequences. This risk is especially high because LTR-retrotransposons share several core proteins with the *Caulimoviridae*, are the most abundant TEs in plant genomes and are also often proximally located to the EVEs [12, 36]. After examining the results obtained from the RPS-BLAST annotation of the consensus sequences, we found no evidence for the presence of LTR-retrotransposons (integrase and Gag proteins), DNA transposons (transposase) or other TEs, suggesting the absence of TE-caulimovirid chimeric sequences.

#### Run 2: tracking florendoviruses without prior knowledge of their existence

In a second run, we investigated how accurately and comprehensively CAULIFINDER Branch A would identify and extract florendoviral sequences from a plant genome if reference sequences for this viral genus were not available. For this, Branch A was launched with default parameters as described above but florendoviral sequences were removed from the caulimovirid genome library used as the initial search query and the florendoviral proteins from the “baits” library (except for the BLAST where “baits” was used for classification by best-hit in step 3, see Fig. 2). The resulting output consensus sequences were compared to the florendoviral reference sequences as described above, using sequence clustering followed by alignment. We obtained results similar to those from the first run for VvinAV, VvinBV_compA and VvinDV compA. However, we obtained only truncated versions of VvinBV_compB, VinDV_compB and VvinCV_sc1 (Fig. 3B and Supplementary Fig. 1), and no sequence clustered with VvinCV_sc2. VvinCV is less abundant than other EVEs in the *V. vinifera* genome and there are fewer full length genome copies: average fragment size is only 593 bp [12]. Hence, manual methods to reconstruct the EVE sequences produce much better results than TEdenovo, especially when the proper probe is absent from the reference virus genome library.

The consensus sequences obtained for the two B genomic components (VvinBV_compB and VinDV_compB) were very truncated at their 3′ end when compared with the reference sequences (Fig. 3). Conserved MP and AP domains and a zinc finger motif were found in the VvinBV-compB-sc1 sequence following a search of the CDD database, but a truncated RH1 domain was also identified when this component sequence was manually reassembled and annotated [12]. In VvinDV_compB, only the MP and zinc finger motif were detected. Furthermore, when compared to the florendovirus-filtered reference genome library using tBLASTx, we found that significant hits were only found against the MP domain (Supplementary Fig. 3), reflecting substantial divergence of the other component B protein domains. As a result, in the initial tBLASTx comparison of the caulimovirid genome library to the *V. vinifera* genome, the component B copies were only detected in positions corresponding to the MP domain, which is located at the 5′ end of the conceptually linearized component sequence. Subsequent extension of the MP sequence in both directions by 2 kbp, which is the default value for the pipeline, was not sufficient to retrieve the entire component B sequences, resulting in the presence of only truncated copies in the sub-genome database.

#### Run 3: chasing for component B

Considering the results obtained in “run 2”, we hypothesized that increasing the extension size around merged hits in step 1 would result in a better representation of full length component B sequences in the sub-genome database. CAULIFINDER Branch A was repeated with the same parameters and libraries as in “run 2” except that a 5 kbp extension on the merged hit loci was applied instead of the default of 2 kbp. As hypothesized, this modification resulted in improved lengths of component B consensus sequences (Fig. 3C and Supplementary Fig. 1). In parallel, we noticed an increase in the number of sequences generated and a greater genome coverage (Table 1).

### Classification of the caulimovirid EVEs using Branch B

**Fig. 4:**
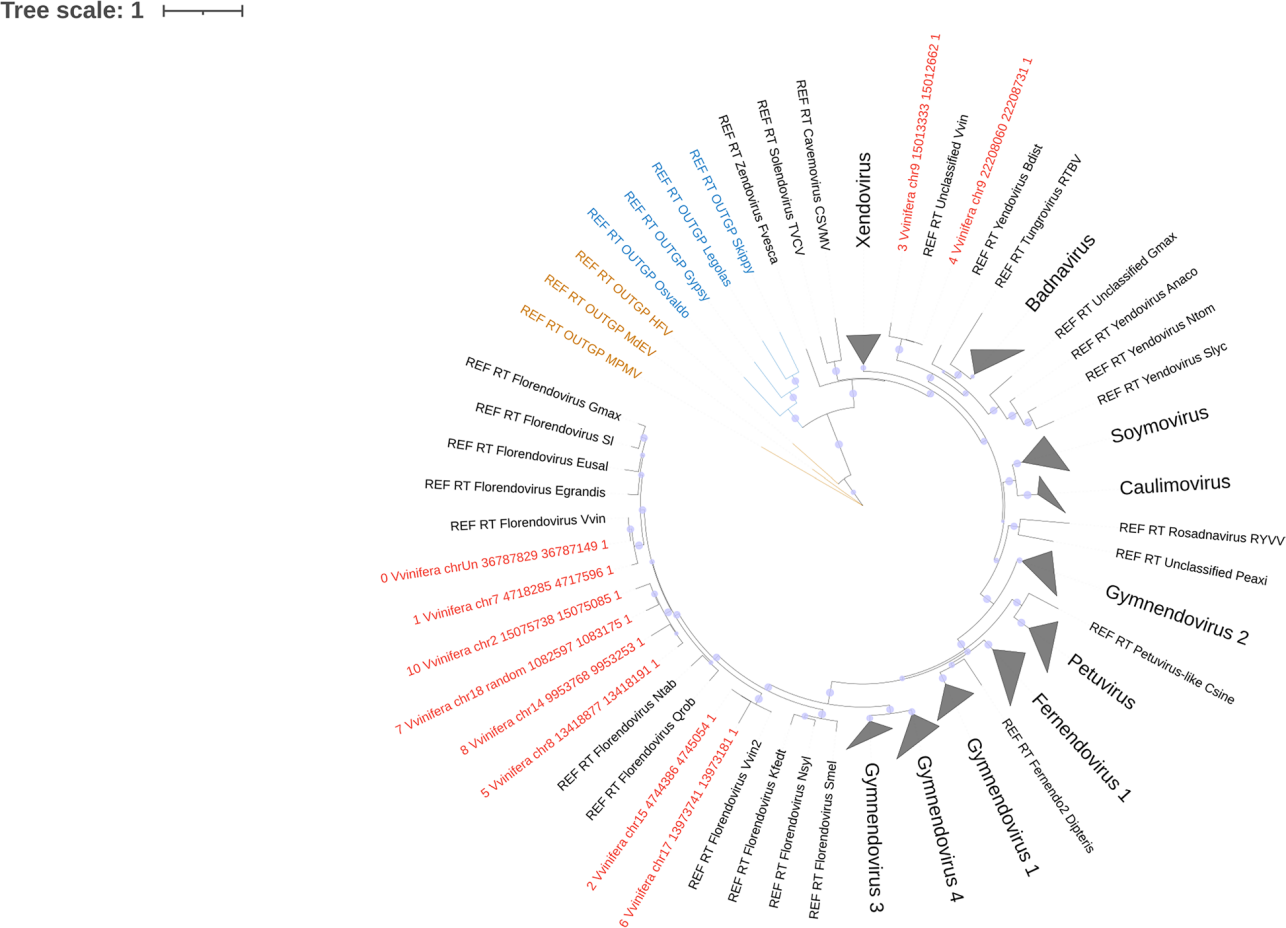
Caulimovirid endogenous viral element diversity in *Vitis vinifera*. Phylogenetic tree of reverse transcriptase domains built from the Newick file obtained from CAULIFINDER Branch B applied on the *V. vinifera* PN40024 genome. All caulimovirid, *Gypsy* and retroviral reference sequences contain the tag ‘REF” in their label. Branches are colored as follows: *Retroviridae* (brown), *Gypsy* elements (blue), *Caulimoviridae* (black) and Branch B representative sequences (red). Several clades have been collapsed for ease of visualization. The reference RT from Vitis endovirus is indicated as “Unclassified Vvin”. Bootstrap values above 70% are highlighted using purple disks in the branch nodes

To benchmark CAULIFINDER Branch B, the *V. vinifera* genome database described above was used. We removed the florendoviral RT probes from the default search library and from the library used to filter out false positives in step 1, but not from the library used in step 3 for constructing the phylogenetic trees. In step 1, Branch B detected 3833 loci with significant matches to the caulimovirid RT probes. These sequences contained 1786 ORFs coding for proteins with a minimum size of 200 aa, of which 84 were retained based on their best BLAST hit against caulimovirid RT sequences. In the second step, the conceptually translated protein sequences were clustered on the basis of similarity, using an 80% aa identity threshold, resulting in ten groups. One representative sequence per group was retained. All representative sequences passed the filtering stages in steps 2 and 3 based on MSA quality assessment with trimAl, resulting in 10 candidate caulimovirid RT protein sequences retained to produce the final alignment in step 3. The output phylogenetic tree showed that CAULIFINDER Branch B succeeded in detecting eight RT sequences clustering in the florendovirus clade, as well as two others that grouped with Vitis endovirus (Fig. 4). The main output files of this run are available in the supplementary dataset.

## Conclusions

This paper describes CAULIFINDER, a package dedicated to the detection of caulimovirid EVEs in plant genomes. CAULIFINDER uses two complementary workflows: Branch A produces a library of consensus sequences, whereas Branch B addresses phylogeny and classification. We have developed CAULIFINDER with three main applications in mind, the first aimed at those studying genome biology, who will benefit from more accurate genome annotations reflecting the presence of caulimovirid EVEs. The second application is relevant to paleovirology, since CAULIFINDER will help unravel cryptic and/or ancient host-virus interactions and inform the evolution of the *Caulimoviridae* over unprecedented timescales. The final application is in the field of plant pathology, as some caulimovirid EVEs retain replication competency and can give rise to new infections. CAULIFINDER will accelerate the discovery of these replication-competent EVEs.

When used with default parameters and libraries, CAULIFINDER Branch A is calibrated to perform sensitive and specific genome annotation for any known genus of the *Caulimoviridae*. For exploratory use, search parameters can be relaxed but at the cost of specificity, since the output library may become contaminated with sequence chimeras or non-target sequences such as those of LTR-retrotransposons: manual curation is recommended in this case. In these instances, the summary table could be examined for the presence of protein domains that are not normally associated with the *Caulimoviridae*. Furthermore, [38] one could compare each consensus-encoded ORF to a comprehensive TE protein library supplemented with caulimovirid ORFs available in the “baits” library using BLASTp to assess if any ORF has best hit against a TE protein, or compare each consensus to a TE sequence library from the species of interest using BLASTn. Any warning could be checked further to confirm chimerism or the concerned consensus could be directly discarded.

Branch A is most efficient if the virus genera expected to be present in a given plant genome are represented in the library of reference genomes used as the initial query. So far, this library encompasses representative sequences from all the genera recognized by the ICTV as well as consensus sequences reconstructed from a diversity of OTUs detected across 62 land plant species, including angiosperms, gymnosperms and ferns [7] (Supplementary Table 1). Owing to a historical sampling bias, the diversity of caulimovirid genera that associate with angiosperms is probably comprehensively represented in this library while many more cryptic genera likely remain to be discovered in other plant clades. Therefore, using the current library, the sensitivity of Branch A should be higher with angiosperm than non-angiosperm species. Nevertheless, the level of conservation between conserved protein domains across caulimovirid genera allows in principle to produce satisfactory results for “canonical” caulimovirid species using Branch A, even without a cognate reference genome in the library, as experienced for florendovirus component A sequences during the benchmark described in this work. The main limitation of CAULIFINDER Branch A that was observed is a decrease in sensitivity for the detection of component B sequences of florendovirus bipartite genomes, which are highly atypical of the *Caulimoviridae*. By running Branch B, users can easily compare the diversity of caulimovirid EVEs present in a given genome to that represented in the CAULIFINDER libraries. All CAULIFINDER librarieswill be updated on a regular basis in the dedicated dataverse (10.57745/ADFNMB) to reflect new discoveries and updates of the taxonomy of viruses.

Considering that Branch A searches for repetitive elements at the genome level, it is recommended to use it only with genome assemblies. It is worth noting that sequence decay accumulate over time in EVEs following integration, causing repetitiveness to be lost gradually. This is likely to affect the sensitivity of Branch A. Branch B scans for RT domain diversity and can be launched on genome assemblies, but it is also adapted for mining transcriptomic datasets (i.e. assembled transcriptomes). Branch B comprises a translation step, therefore it is not recommended to use it on uncorrected long DNA sequencing reads produced from PacBio and ONT technologies since sequencing errors could interrupt RT ORFs.

In summary, CAULIFINDER is a robust package that can be easily implemented by most scientists with basic bioinformatics skills to annotate caulimovirid EVEs in plant genomes and to collect valuable sequences to support evolutionary studies. CAULIFINDER could also be easily adapted for the discovery of endogenous retroviruses and geminivirids.

## Availability and requirements

Project name: CAULIFINDER

Project home page: https://forgemia.inra.fr/urgi-anagen/event_caulifinder

Operating system(s): Platform independent, Docker project at https://forgemia.inra.fr/urgi-anagen/caulifinder_docker, Docker image at https://hub.docker.com/r/urgi/docker_caulifinder

Caulifinder libraries: 10.57745/ADFNMB

Programming language: Python and Bash

Other requirements: Docker

License: MIT

Any restrictions to use by non-academics: none

## Supplementary Information

**Additional file 1.**

**Additional file 2: Supplementary Figure 1.** Graphical overview of the multiple sequence alignments obtained for each cluster containing Florendovirus and Vitis endovirus reference sequences, for each of the Branch A runs. Concatemer sequences have not been filtered. The alignments were obtained using MAFFT with the ginsi and leave gappy regions 0.8 settings and visualized in the overview window of the Jalview program with nucleotide colours. For Vitis endovirus, only the results of run 1 are shown.

**Additional file 3: Supplementary Figure 2.** Sequence dot plots for two examples of concatemers detected in the output of Branch A run1. For each concatemer, the dot plot was generated against the query sequence itself and against all the other sequences of the cluster it belongs to, except concatemers. Forward and reverse hits are indicated as green and red lines, respectively. Coverage density is indicated on the axes with the same color code. The dot plots were produced using the YASS web server (https://bioinfo.lifl.fr/yass/yass.php).

**Additional file 4: Supplementary Figure 3.** Graphical output of tBLASTx sequence comparison using VvinBV_compBsc1 (A) and VinDV_compBsc1 (B) against the Caulimoviridae genome library without Florendovirus representatives.

## Data Availability

The supplementary dataset contains the following items: • Supplementary Figures • Supplementary Table 1 • Branch A run 1 summary archive • Branch B summary archive It is available at https://figshare.com/projects/Caulifinder/143532
