# Supplementary material for: CAULIFINDER: a pipeline for the automated detection and annotation of caulimovirid endogenous viral elements in plant genomes
Source: Mob DNA. 2022 Dec 3;13:31. doi: 10.1186/s13100-022-00288-w (PMC9719215; doi:10.1186/s13100-022-00288-w)

CAULIFINDER: a pipeline for the automated detection and annotation of caulimovirid endogenous viral elements in plant genomes

Supplementary Figure 1

Graphical overview of the multiple sequence alignments obtained for each cluster containing Florendovirus and Vitis endovirus reference sequences, for each of the Branch A runs. Concatemer sequences have not been filtered. The alignments were obtained using MAFFT with the ginsi and leave gappy regions 0.8 settings and visualized in the overview window of the Jalview program with nucleotide colours. For Vitis endovirus, only the results of run 1 are shown.

run 1 – V. vinifera – cluster 1 (Florendovirus VvinAV & VvinBV)

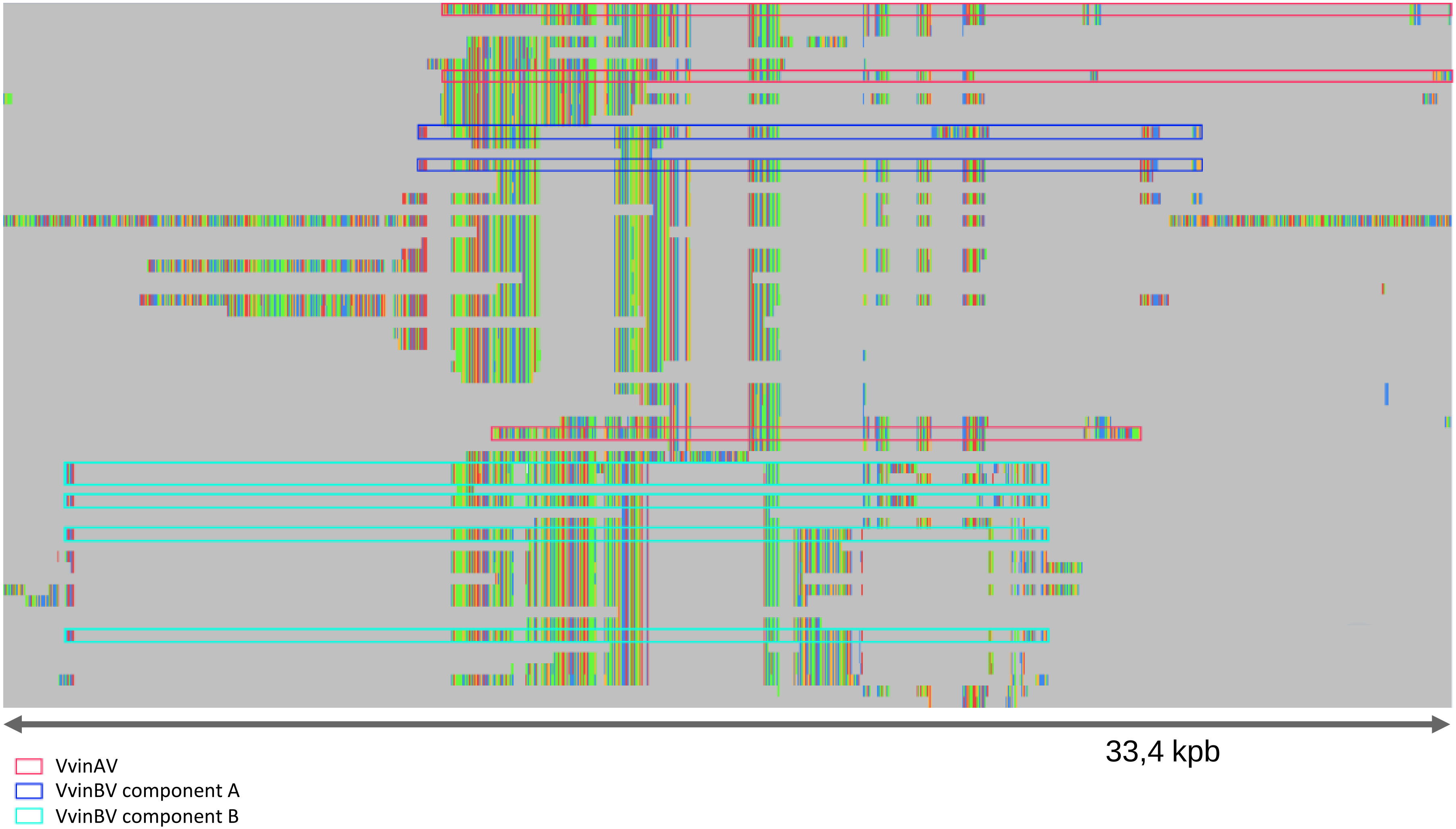

run 1 – V. vinifera – cluster 2 (Florendovirus VvinDV)

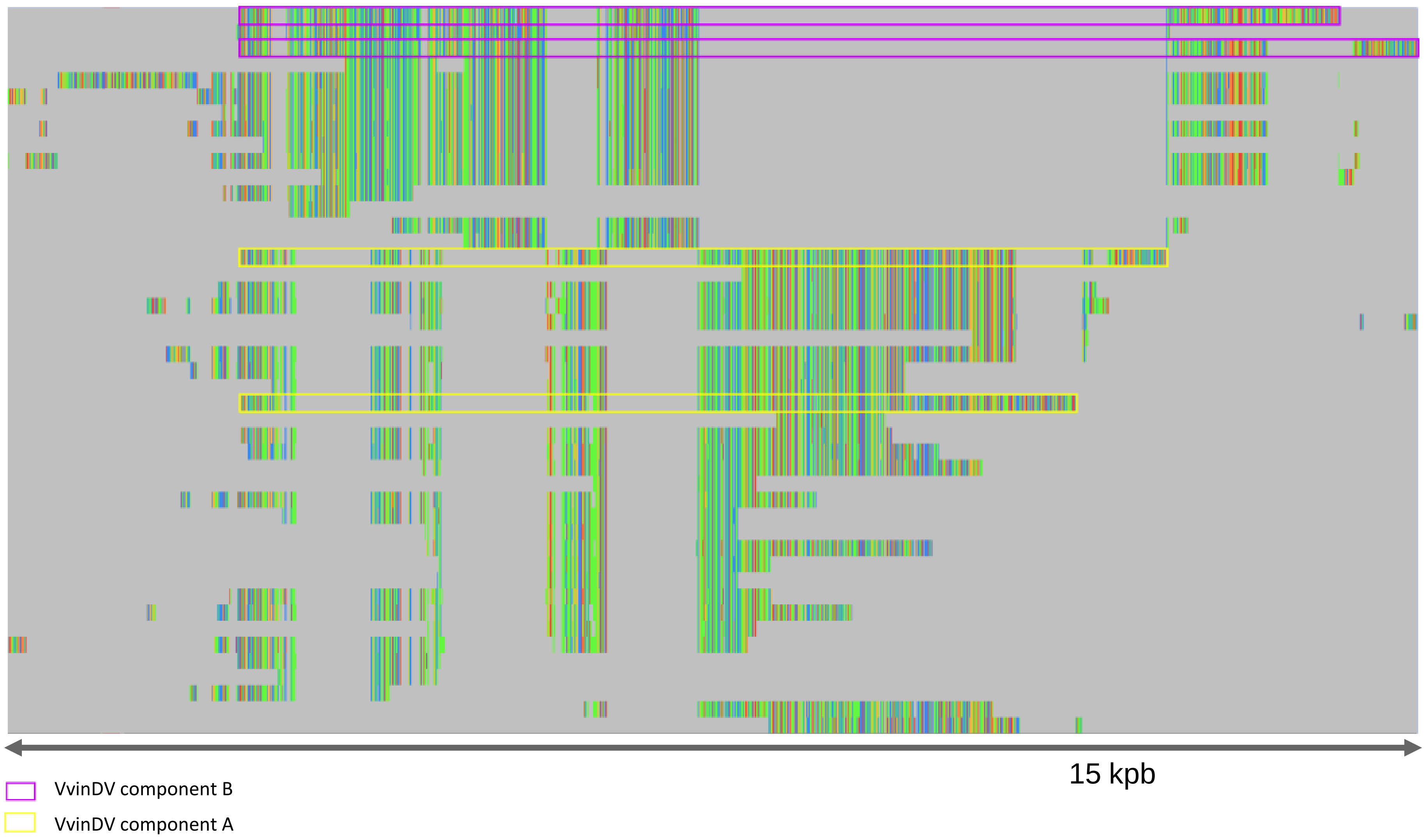

run 1 – V. vinifera – cluster 3 (Florendovirus VvinCV)

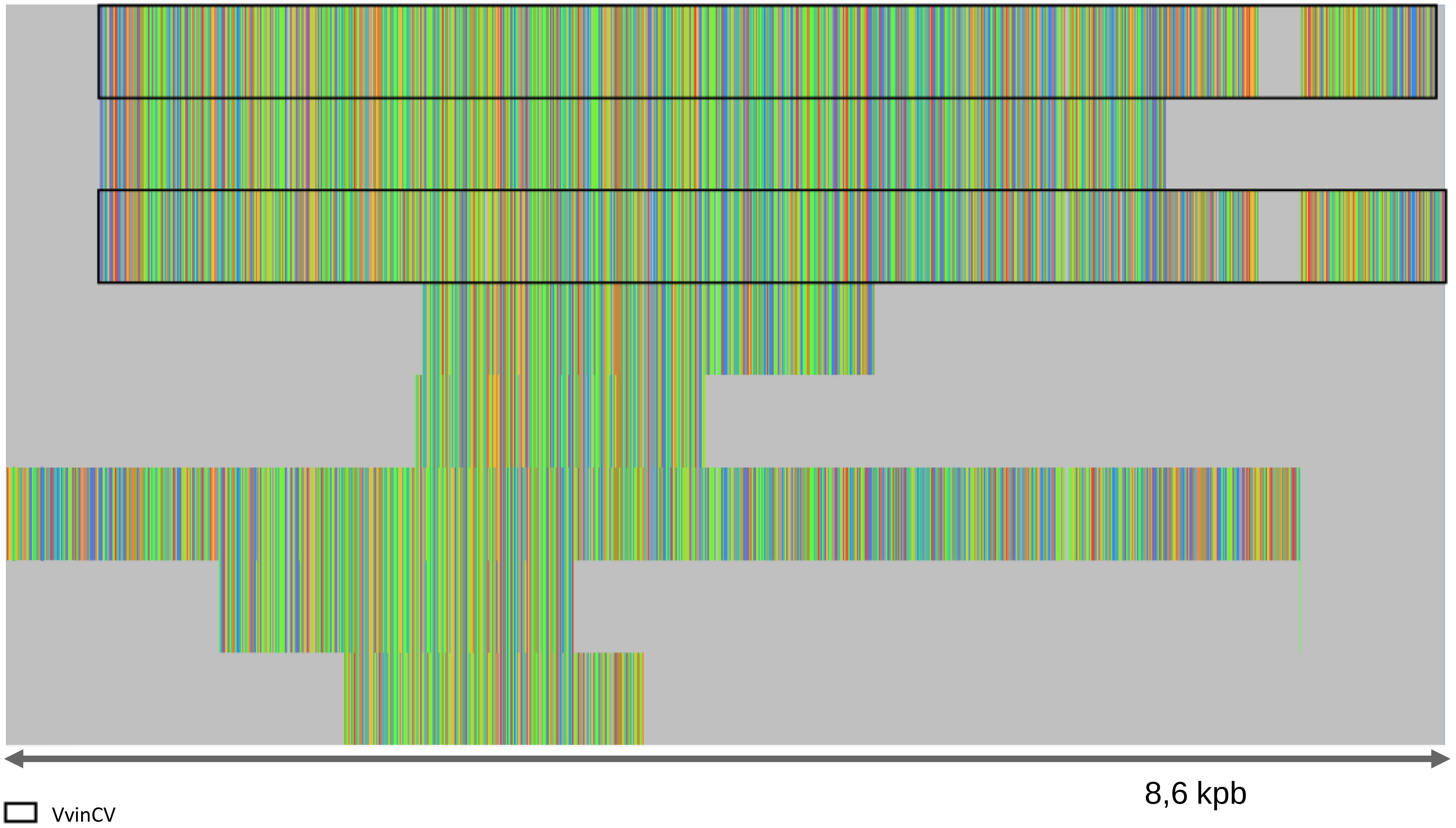

run 1 – V. vinifera – cluster 4 (Vitis endovirus)

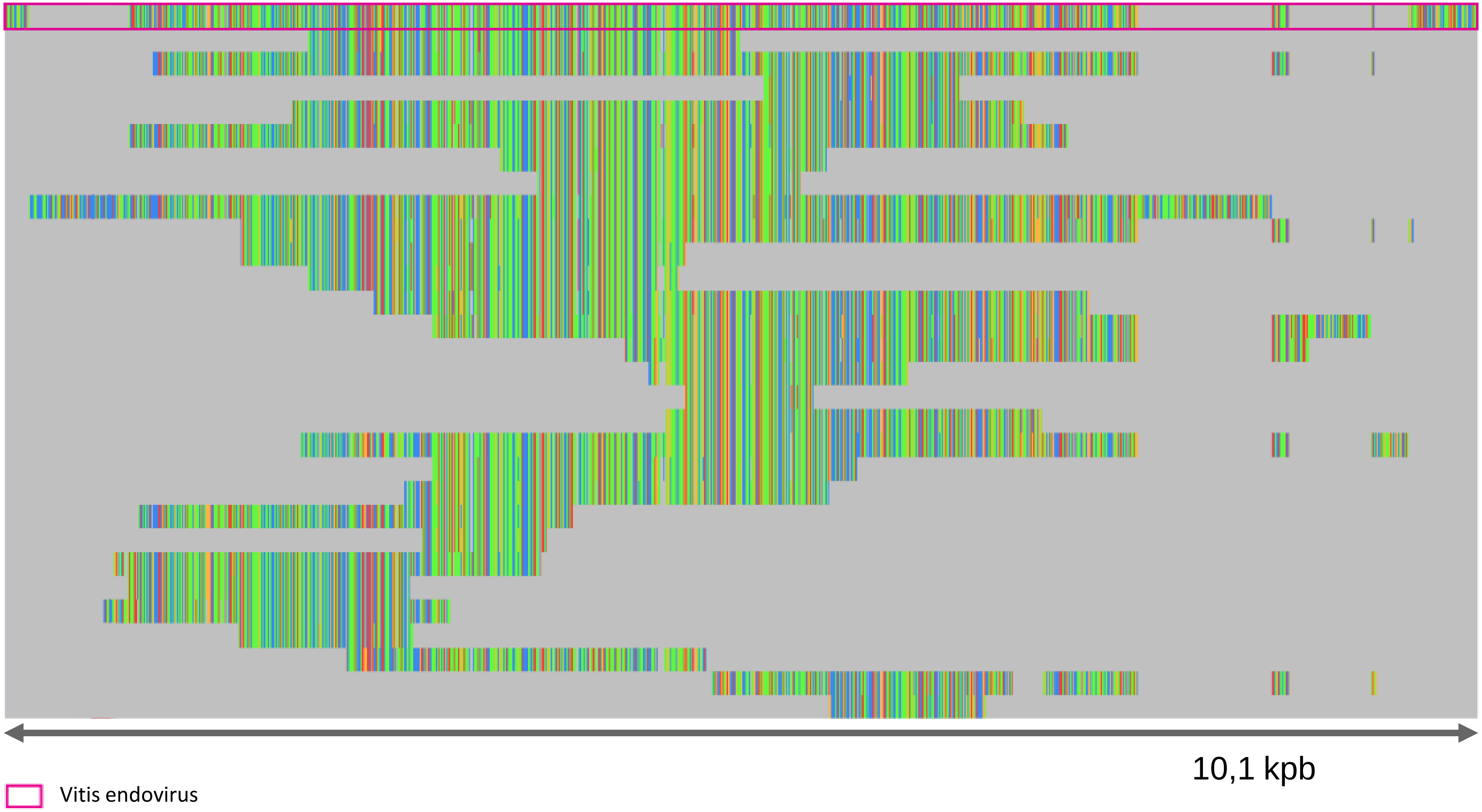

run 2 – V. vinifera – cluster 1 (Florendovirus VvinAV & VvinBV)

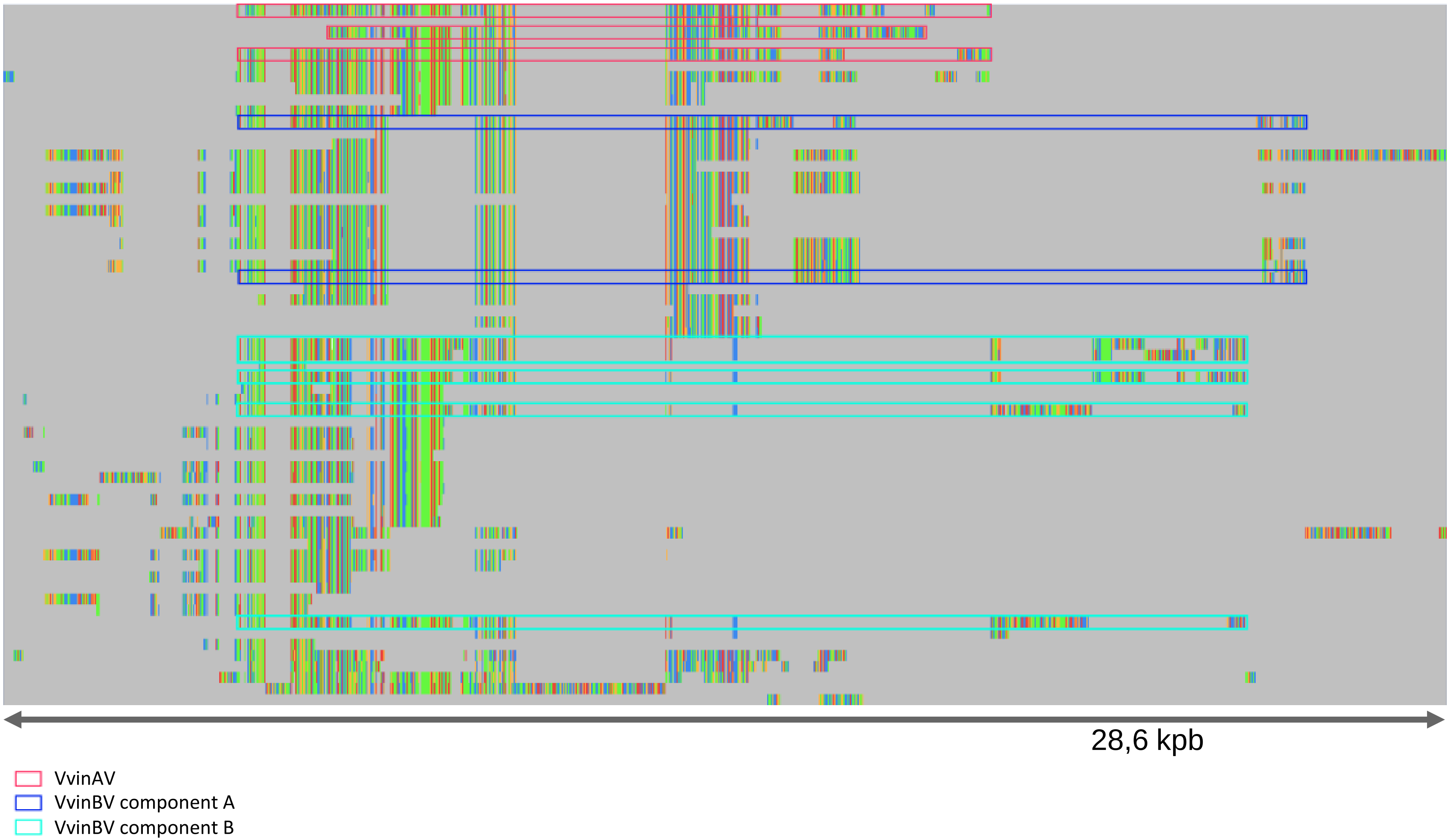

run 2 – V. vinifera – cluster 2 & 3 (Florendovirus VvinDV compA and CompB, respectively)

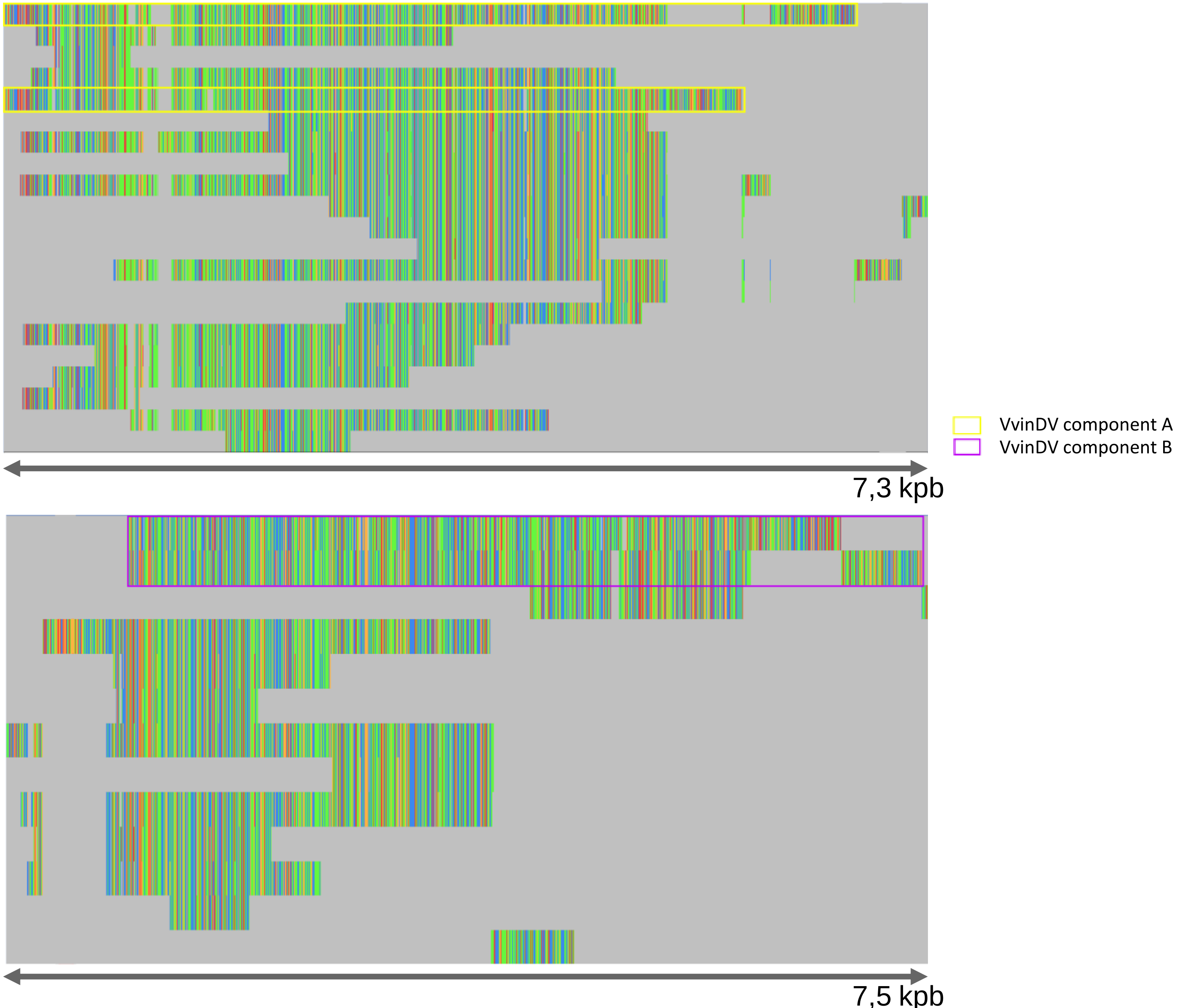

run 2 – V. vinifera – cluster 4 (Florendovirus VvinCV)

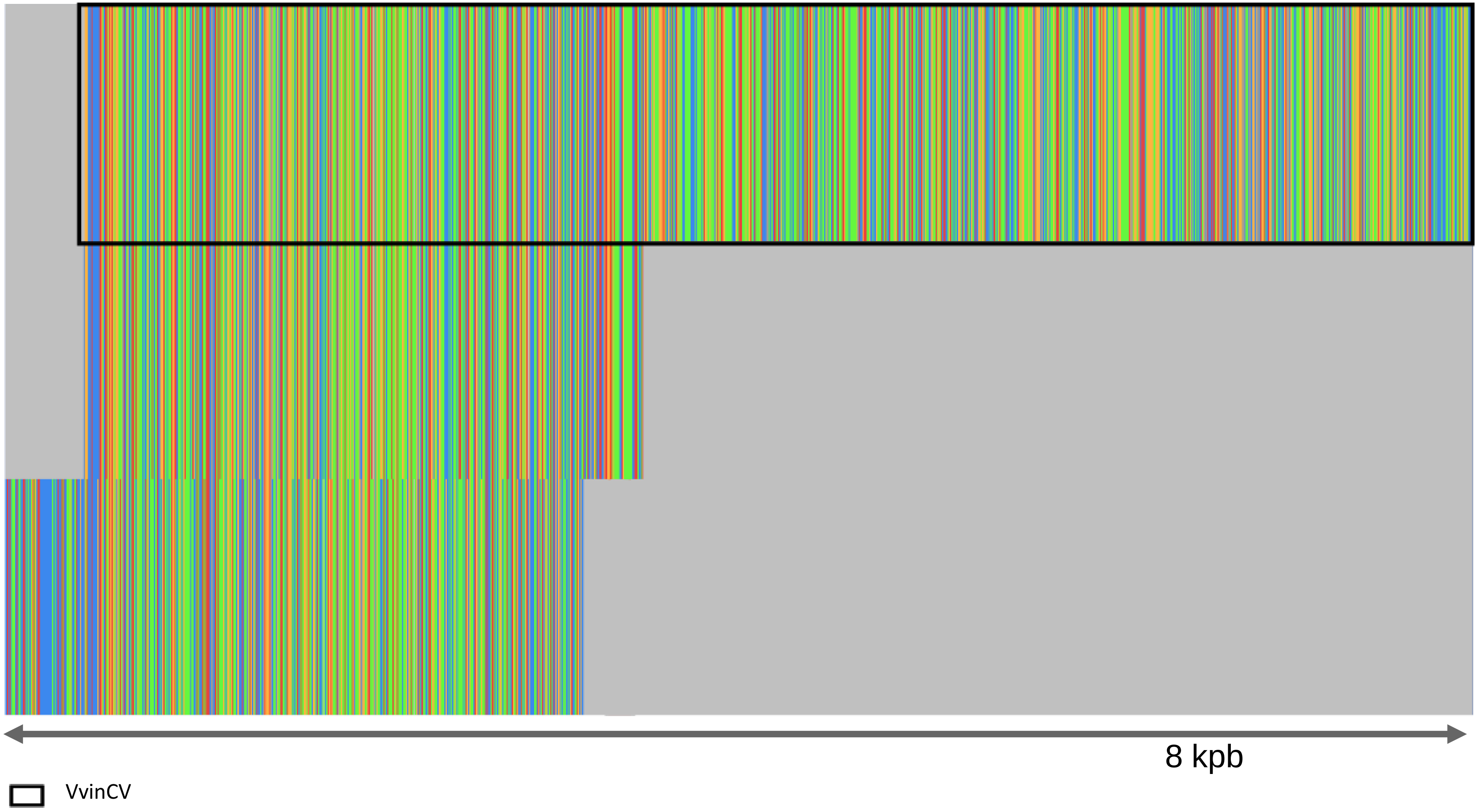

run 3 – V. vinifera – cluster 1 (Florendovirus VvinAV & VvinBV)

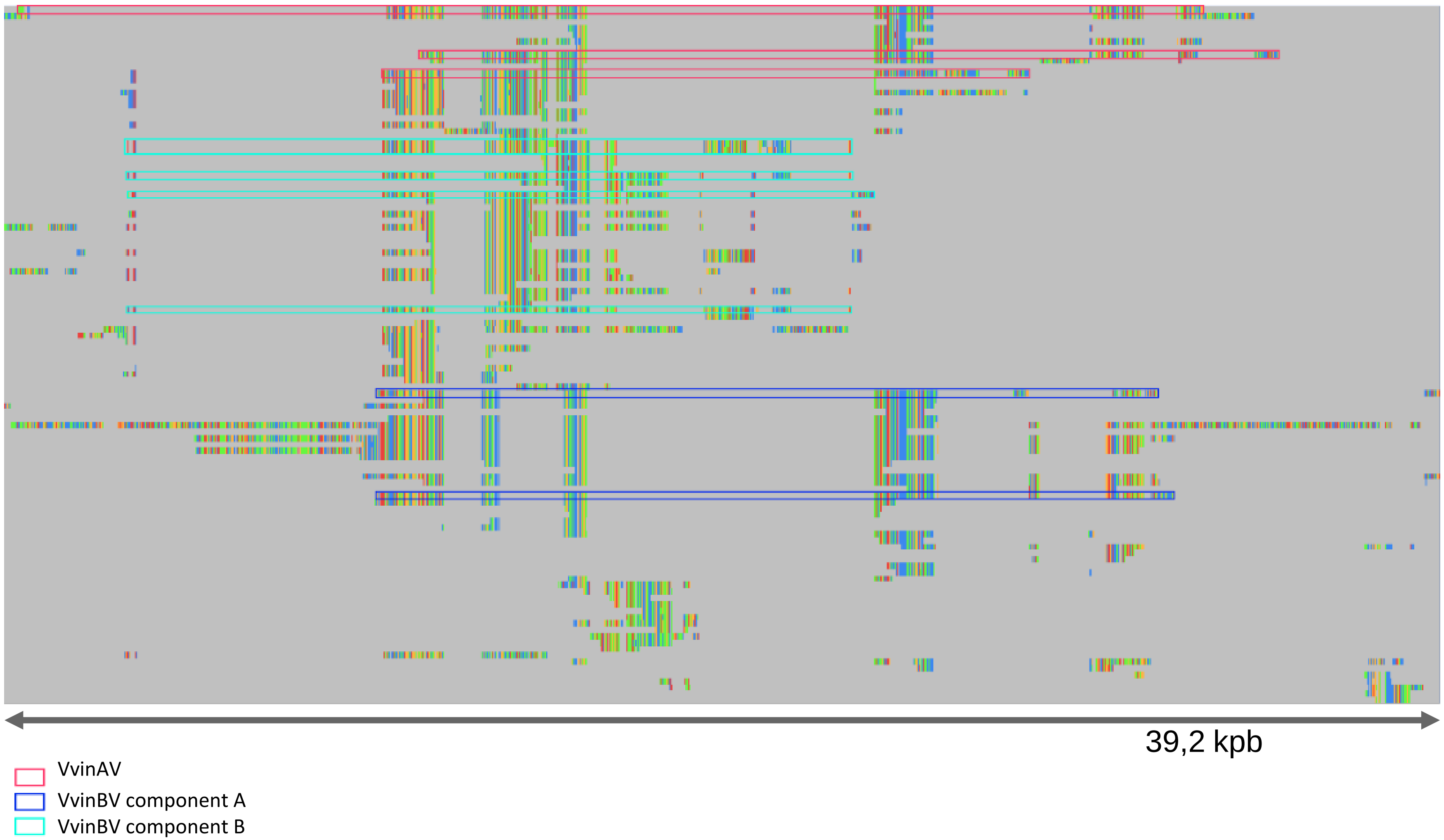

run 3 – V. vinifera – cluster 2 (Florendovirus VvinDV)

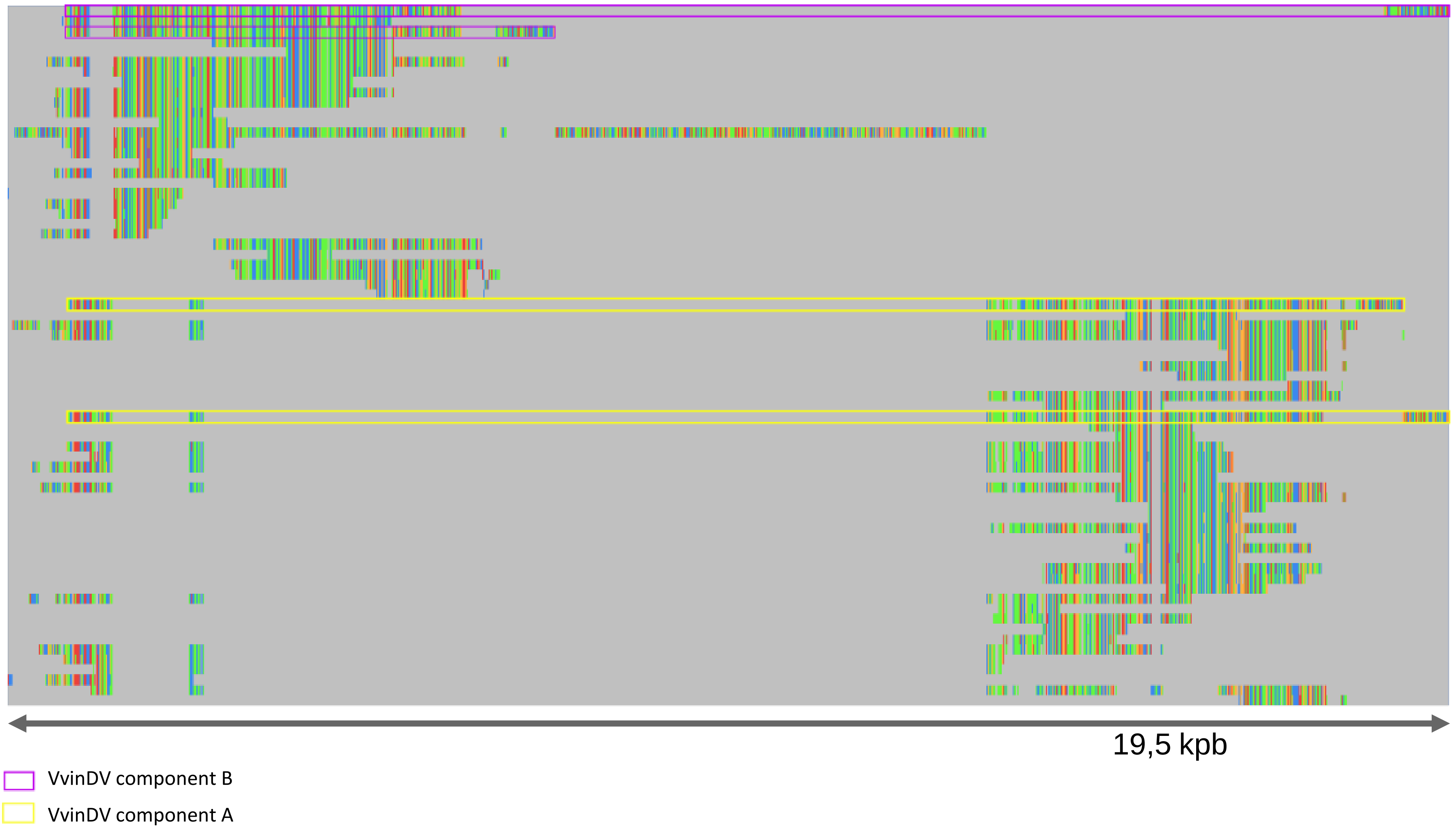

run 3 – V. vinifera – cluster 3 (Florendovirus VvinCV)

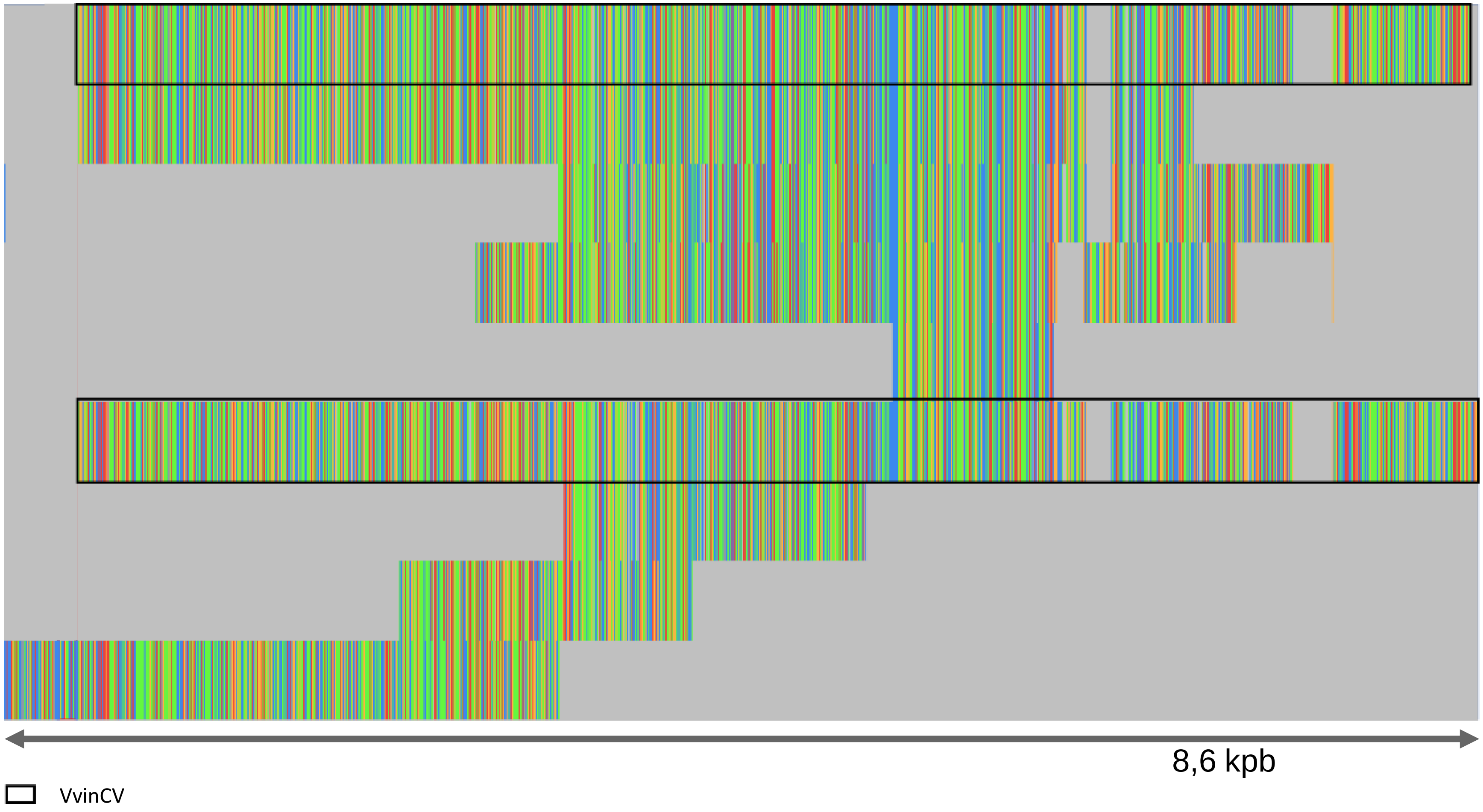

Supplement: Supplementary file 2 — Additional file 2: Supplementary Figure 1. Graphical overview of the multiple sequence alignments obtained for each cluster containing Florendovirus and Vitis endovirus reference sequences, for each of the Branch A runs. Concatemer sequences have not been filtered. The alignments were obtained using MAFFT with the ginsi and leave gappy regions 0.8 settings and visualized in the overview window of the Jalview program with nucleotide colours. For Vitis endovirus, only the results of run 1 are shown. [file 13100_2022_288_MOESM2_ESM.pdf]
