# Supplementary material for: CAULIFINDER: a pipeline for the automated detection and annotation of caulimovirid endogenous viral elements in plant genomes
Source: Mob DNA. 2022 Dec 3;13:31. doi: 10.1186/s13100-022-00288-w (PMC9719215; doi:10.1186/s13100-022-00288-w)

# CAULIFINDER: a pipeline for the automated detection and annotation of caulimovirid endogenous viral elements in plant genomes

## Supplementary Figure 2

Sequence dot plots for two examples of concatemers detected in the output of Branch A run1. For each concatemer, the dot plot was generated against the query sequence itself and against all the other sequences of the cluster it belongs to, except concatemers. Forward and reverse hits are indicated as green and red lines, respectively. Coverage density is indicated on the axes with the same color code. The dot plots were produced using the YASS web server (https://bioinfo.lifl.fr/yass/yass.php)

# Recon_10_Map_20 against itself

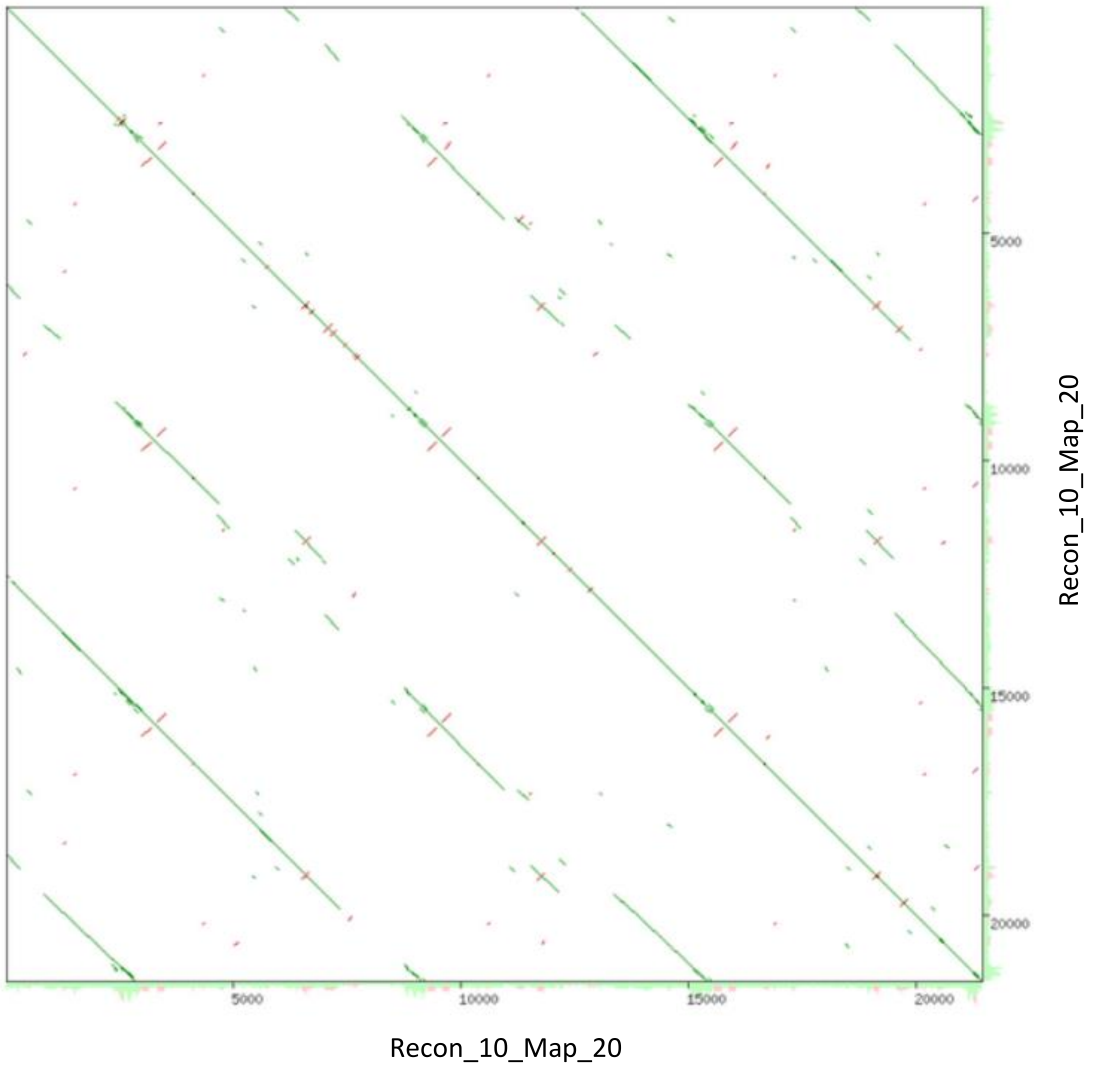

# Recon_10_Map_20 against cluster

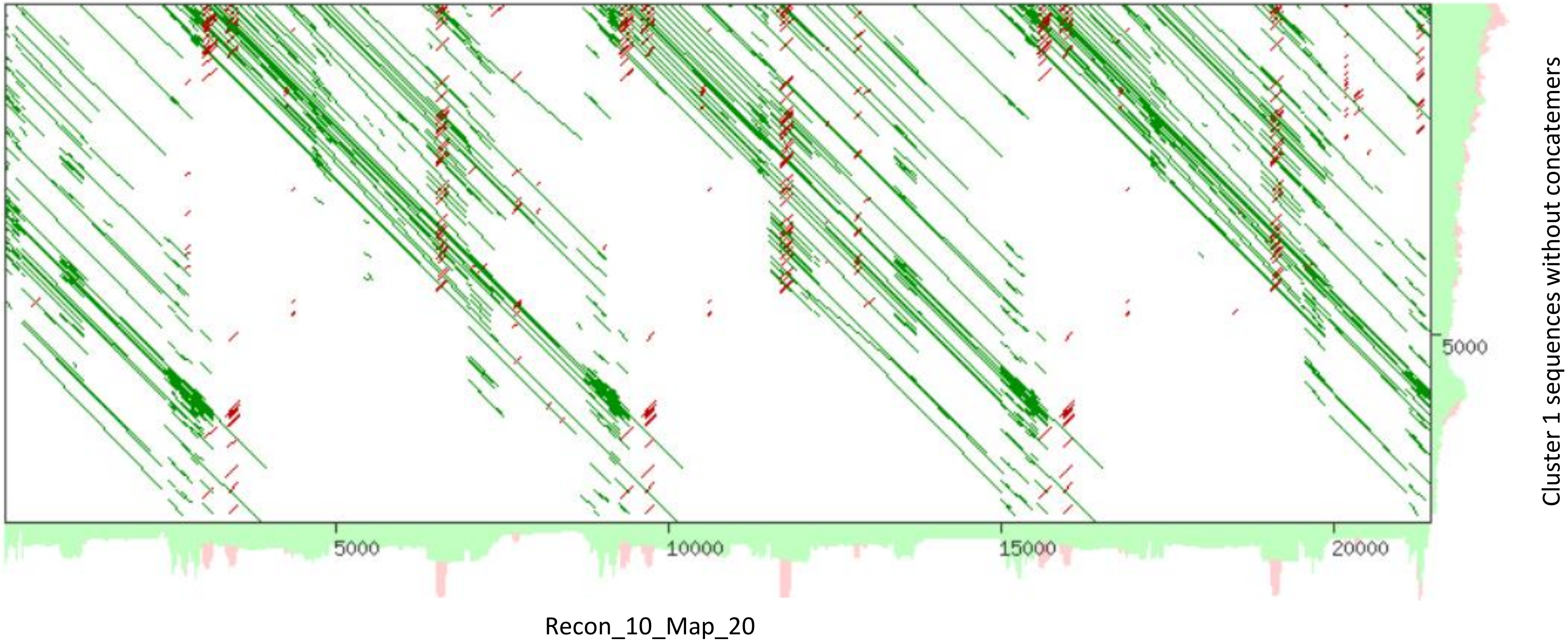

# Grouper_132_Map_6 against itself

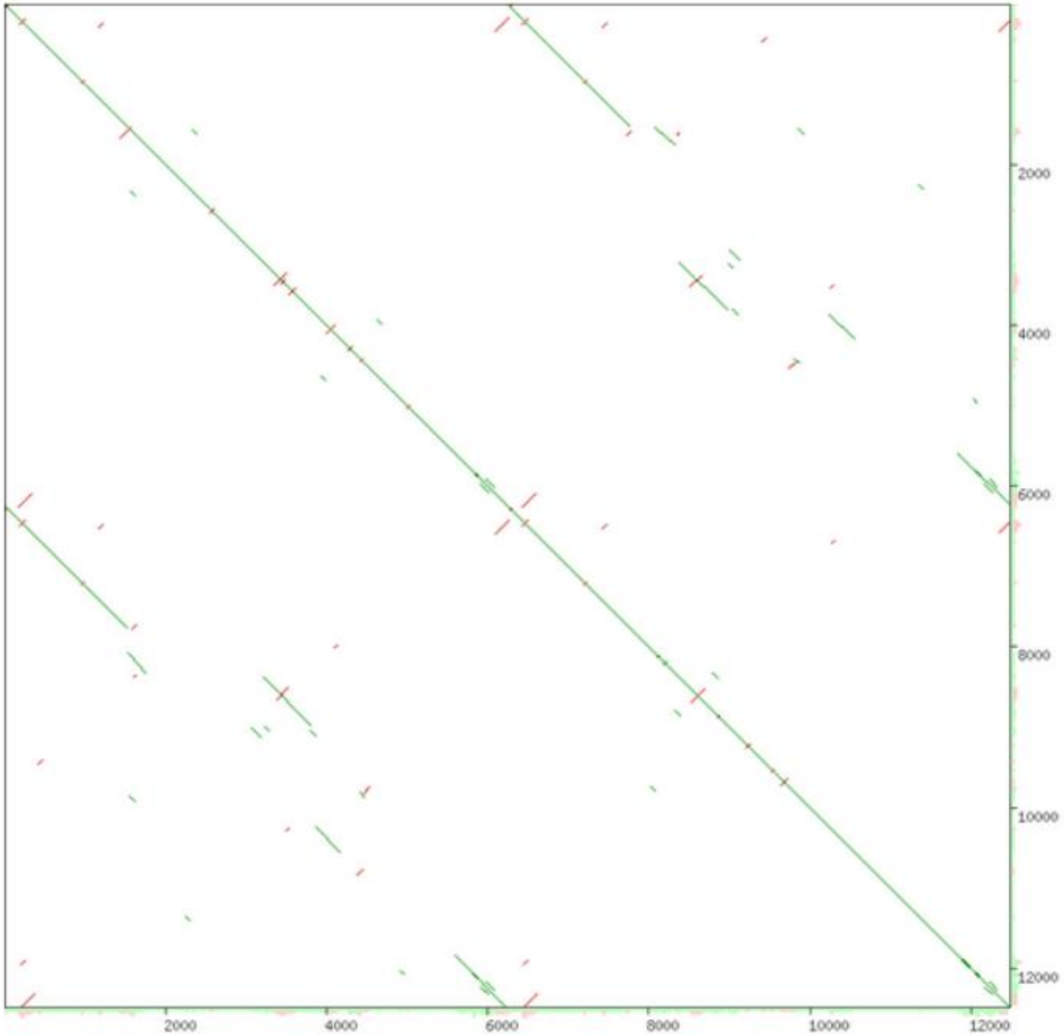


Grouper_132_Map_6

Grouper_132_Map_6

# Grouper_132_Map_6 against cluster

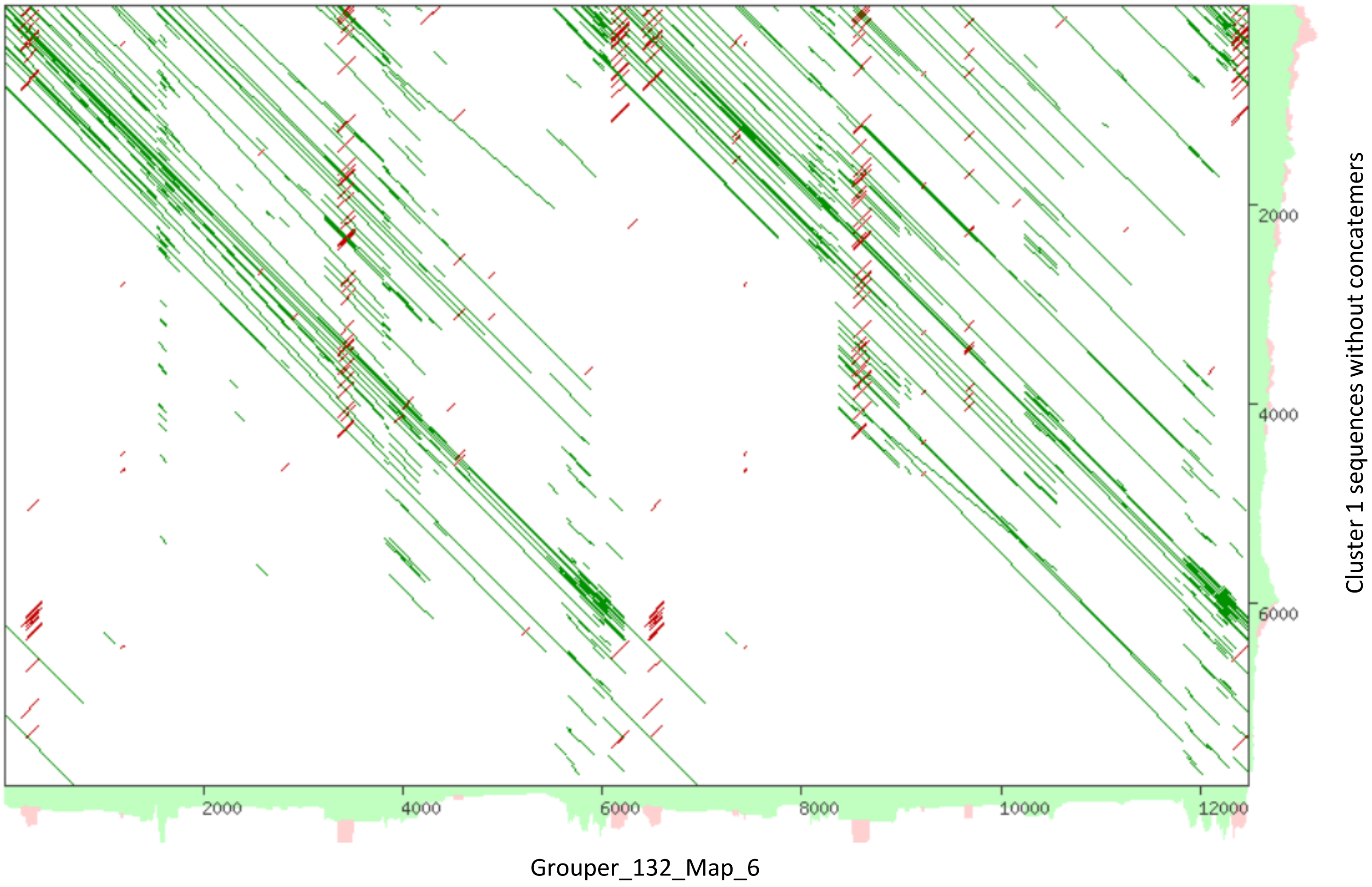

Supplement: Supplementary file 3 — Additional file 3: Supplementary Figure 2. Sequence dot plots for two examples of concatemers detected in the output of Branch A run1. For each concatemer, the dot plot was generated against the query sequence itself and against all the other sequences of the cluster it belongs to, except concatemers. Forward and reverse hits are indicated as green and red lines, respectively. Coverage density is indicated on the axes with the same color code. The dot plots were produced using the YASS web server (https://bioinfo.lifl.fr/yass/yass.php). [file 13100_2022_288_MOESM3_ESM.pdf]
