# Supplementary material for: CAULIFINDER: a pipeline for the automated detection and annotation of caulimovirid endogenous viral elements in plant genomes
Source: Mob DNA. 2022 Dec 3;13:31. doi: 10.1186/s13100-022-00288-w (PMC9719215; doi:10.1186/s13100-022-00288-w)
Supplement: Supplementary file 4 — Additional file 4: Supplementary Figure 3. Graphical output of tBLASTx sequence comparison using VvinBV_compBsc1 (A) and VinDV_compBsc1 (B) against the Caulimoviridae genome library without Florendovirus representatives. [file 13100_2022_288_MOESM4_ESM.pdf]

CAULIFINDER: a pipeline for the automated detection and annotation of caulimovirid endogenous viral elements in plant genomes

Supplementary Figure 3

Graphical output of tBLASTx sequence comparison using VvinBV_compBsc1 (A) and VinDV_compBsc1 (B) against the Caulimoviridae genome library without Florendovirus representatives.

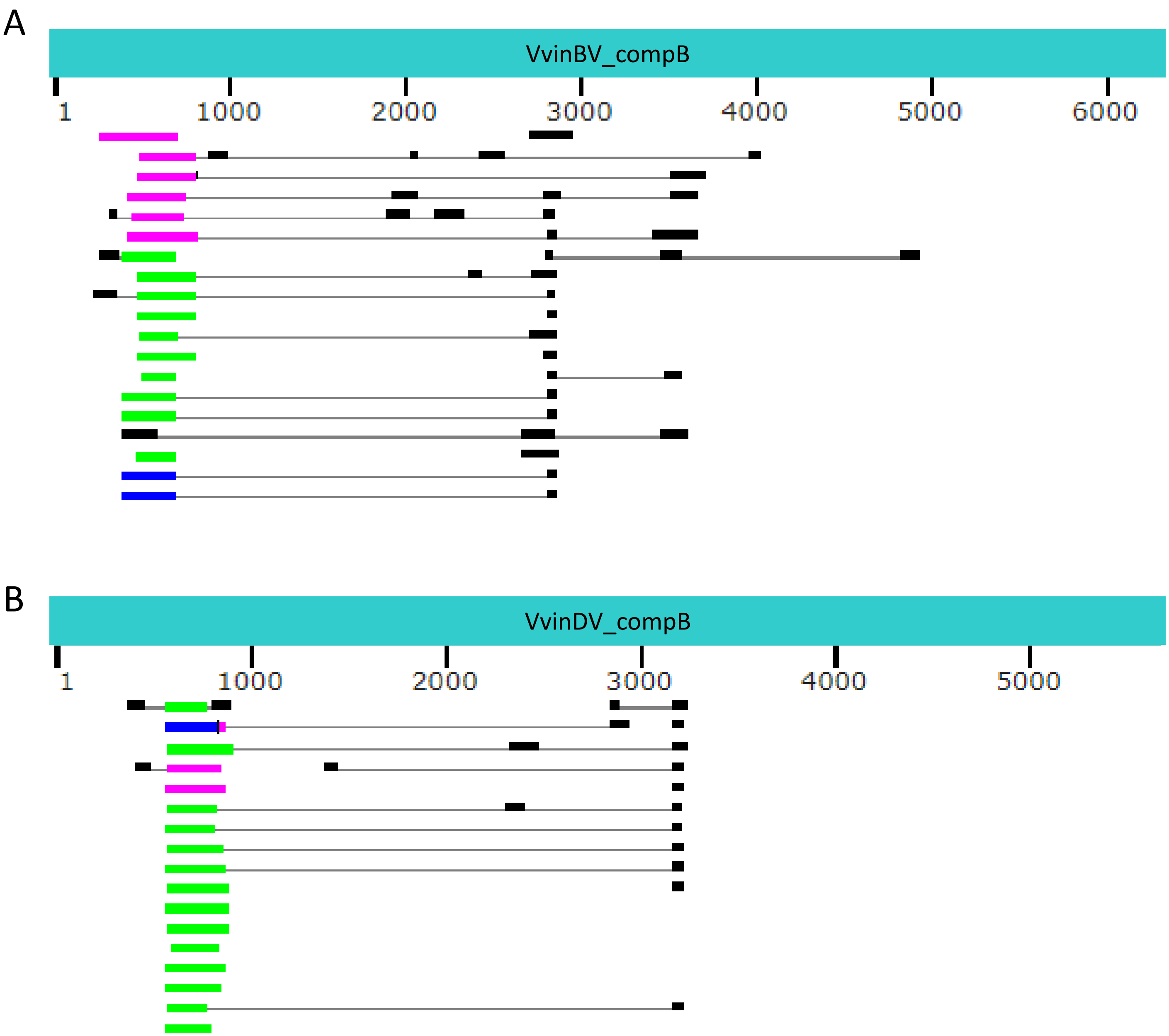
A
VvinBV_compB
1
1000
2000
3000
4000
5000
6000
B
VvinDV_compB
1
1000
2000
3000
4000
5000
